# Genome-Wide Analysis in Brazilians Reveals Highly Differentiated Native American Genome Regions

**DOI:** 10.1093/molbev/msw249

**Published:** 2017-01-18

**Authors:** Josyf C. Mychaleckyj, Alexandre Havt, Uma Nayak, Relana Pinkerton, Emily Farber, Patrick Concannon, Aldo A. Lima, Richard L. Guerrant

**Affiliations:** 1 Center for Public Health Genomics, University of Virginia, Charlottesville, VA; 2 Department of Public Health Sciences, University of Virginia, Charlottesville, VA; 3 Departamento de Fisiologia e Farmacologia, Universidade Federal do Ceará, Fortaleza, Brazil; 4 INCT-Instituto de Biomedicina Universidade Federal do Ceará, Fortaleza, Brazil; 5 Center for Global Health, University of Virginia, Charlottesville, VA; 6 Genetics Institute, University of Florida, Gainesville, FL; 7 Department of Pathology Immunology and Laboratory Medicine, University of Florida, Gainesville, FL

**Keywords:** ancestry, selection, admixture, genetic differentiation, Native American, Brazil.

## Abstract

Despite its population, geographic size, and emerging economic importance, disproportionately little genome-scale research exists into genetic factors that predispose Brazilians to disease, or the population genetics of risk. After identification of suitable proxy populations and careful analysis of tri-continental admixture in 1,538 North-Eastern Brazilians to estimate individual ancestry and ancestral allele frequencies, we computed 400,000 genome-wide locus-specific branch length (LSBL) Fst statistics of Brazilian Amerindian ancestry compared to European and African; and a similar set of differentiation statistics for their Amerindian component compared with the closest Asian 1000 Genomes population (surprisingly, Bengalis in Bangladesh). After ranking SNPs by these statistics, we identified the top 10 highly differentiated SNPs in five genome regions in the LSBL tests of Brazilian Amerindian ancestry compared to European and African; and the top 10 SNPs in eight regions comparing their Amerindian component to the closest Asian 1000 Genomes population. We found SNPs within or proximal to the genes *CIITA* (rs6498115), *SMC6* (rs1834619), and *KLHL29* (rs2288697) were most differentiated in the Amerindian-specific branch, while SNPs in the genes *ADAMTS9* (rs7631391), *DOCK2* (rs77594147), *SLC28A1* (rs28649017), *ARHGAP5* (rs7151991), and *CIITA* (rs45601437) were most highly differentiated in the Asian comparison. These genes are known to influence immune function, metabolic and anthropometry traits, and embryonic development. These analyses have identified candidate genes for selection within Amerindian ancestry, and by comparison of the two analyses, those for which the differentiation may have arisen during the migration from Asia to the Americas.

## Introduction

In the last three decades, Brazil has undergone a rapid transition from low-middle income to a burgeoning high-income country, and this economic development has led to a public health paradox; the diseases of poverty such as infectious diseases and malnutrition, although in decline, still exist (Paim et al. 2011; Victora et al. 2011), but now co-exist with an increasing incidence of “western” lifestyle metabolic diseases (de Carvalho Vidigal et al. 2013). Genetic analysis of susceptibility is an important tool to understand both diseases of poverty and wealth, but to apply genetics to Brazilian sub-populations, an understanding of the structure of the underlying genetic admixture is needed.

Twenty-first century Brazil has emerged as a genetic melting pot of races and ethnic groups reflecting its successive history of conquest, slavery, and migration. Three trans-continental population groups, Europeans, Africans, and native American Indians (Amerindians) substantially contribute to the variable ancestry within Brazil’s population. Although exact estimates differ, there were at least three million indigenous Amerindians in Brazil when Portuguese explorers first landed in Bahia in 1500, but their numbers dropped precipitously during the next centuries through enslavement and forced labor, conflict with the invading colonists, and European-borne disease epidemics, although their numbers have rebounded somewhat since the mid-20th century, to a 2010 census enumeration of 820 thousand (Ricardo and Ricardo 2011) (Fundação Nacional do Índio, http:www.funai.gov.br; last accessed October 13, 2015). In the period after initial discovery by Europeans, migration gradually increased so that by 1,760, approximately 700 thousand Europeans had migrated to Brazil (Venâncio 2000). At the same time, to supplement and ultimately largely replace indigenous Amerindians as a workforce, Africans were imported as slave labor to work on colonial plantations, mostly for sugar production, but also for wood and other agricultural products (Schwartz 1978). African slave trafficking brought to Brazil an estimated total of 4.9 million slaves, 40% of all Africans shipped to the Americas (Bergad 2007).

In comparison to its population size and economic status, relatively little genetic work has been performed on Brazilian populations using genome-wide genetic panels for analysis (Giolo et al. 2012; Kuhn et al. 2012; Kehdy et al. 2015; Lima-Costa et al. 2015). Much of the previous research in Brazil, even during the current genomic era, has used genetic panels with limited numbers of SNPs leading to regionally biased estimates of population genetic parameters with high variance (Cardena et al. 2013; Manta et al. 2013; Vieira et al. 2013; Durso et al. 2014; Ruiz-Linares et al. 2014; Magalhaes da Silva et al. 2015). The majority of the genetics research has been aimed at estimating admixture and correlation with self-reported or socially perceived race groups, with little focus on genetic variants influencing disease risk (Guindalini, Colugnati, et al. 2010; Guindalini, Lee, et al. 2010; Suarez-Kurtz 2010; Suarez-Kurtz et al. 2012). As a necessary precursor to our work to identify genetic factors underlying infant growth and development, we undertook a genome-wide and locus-specific analysis of admixture in our study populations recruited in North-Eastern Brazil, centered on the capital city of Ceará state, Fortaleza, but also including participants from neighboring Paraíba, Pernambuco, and Piauí states. We developed improved estimates of locus-specific admixture and allele frequencies, and used these to identify highly differentiated outlier SNPs in the Amerindian component of the Brazilian ancestry as candidates for SNPs and genes under selection pressure.

## Results

**Fig. 1.**
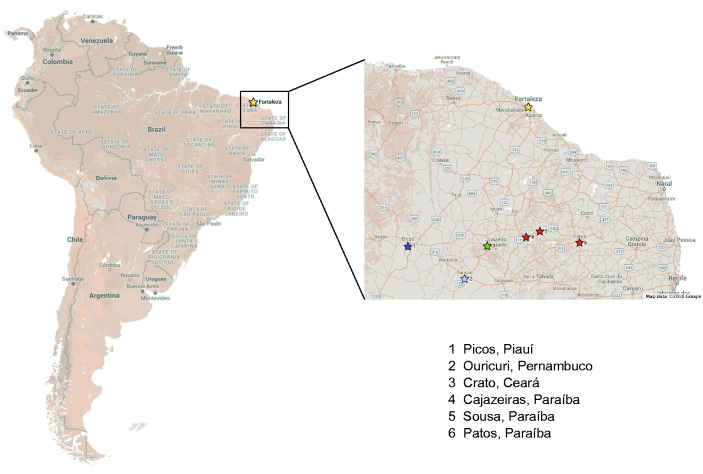
Geographical Map of Brazil showing the location of Fortaleza, the capital city of Ceará state, and other study centers. The inset shows the location of study recruitment centers in the North-Eastern region of Brazil. The location of Fortaleza is indicated by the yellow star icon, and other state color-coded locations are: Picos (Piauí—dark blue); Ouricuri (Pernambuco—light blue); Crato (Ceará—green); Cajazeiras, Sousa and Patos (Paraíba—red). For scale, the distance from Fortaleza to Picos (1.) or from Fortaleza to Ouricuri (2.) is approximately 300 miles.

We genotyped 2,010 DNA samples from six Brazil studies on the Affymetrix Axiom LAT-1 Latin American Array. These samples were drawn from six studies in the North-Eastern region of Brazil, centered on the city of Fortaleza, Ceará state, but also including six other towns or cities (fig. 1). After quality control, we were left with 1,538 samples unrelated to second degree, and 755,801 SNPs (table 1). The detailed quality control steps and results are available in supplementary table S1, Supplementary Material online.

**Table 1.** The Six North-Eastern Brazil Studies and Results of the Genome-Wide Genotyping Quality Control (QC)

	Gonçalves Dias	Mal-ED Birth	Mal-ED Case Control	Recodisa Case Control	PU Zinc-Arginine Trial	PU Zinc Vitamin A Trial	Total
Study Type^a^	Birth Cohort	Birth Cohort	Prospective Case-Control	Prospective Case-Control	Randomized Clinical Trial	Randomized Clinical Trial	
Location	Fortaleza, Gonçalves Dias Favela	Fortaleza	Fortaleza, IPREDE	6 Cities in 4 North-Eastern States	Fortaleza, Parque Universitário	Fortaleza, Parque Universitário	
Enrollment	1989–1993	2010–2014	2010–2014	2010–2014	2006–2010	2000–2006	
Samples Genotyped	172	300	368	1044	126	109	2,119
Samples Post-Genotyping QC	110	276	336	658	95	63	1,538

SNP QC^b^	All Cohorts: SNPs						
Total SNPs on Affymetrix Axiom LAT-1 Array 4	818,154						
Affymetrix SNP QC: SNPs dropped	62,353						
SNPs Remaining	755,801						
Call Rate < 99% + MAF^c^ < 5%	−345,629						
SNPS Remaining	410,172						

a The top half of the table shows the number of DNA samples genotyped by study and the number remaining after genome-wide genotyping QC.

b The bottom half of the table shows the initial total number of SNPs on the Affymetrix array used and the number remaining after QC. The same SNP results pertain to all studies and are only shown once for clarity.

c MAF: Minor Allele Frequency.

### Unsupervised Principal Component Analysis Identified the Latent Major Axes of Trans-Continental Ancestry Defined by Genetic Variation within the Brazil Samples

**Fig. 2.**
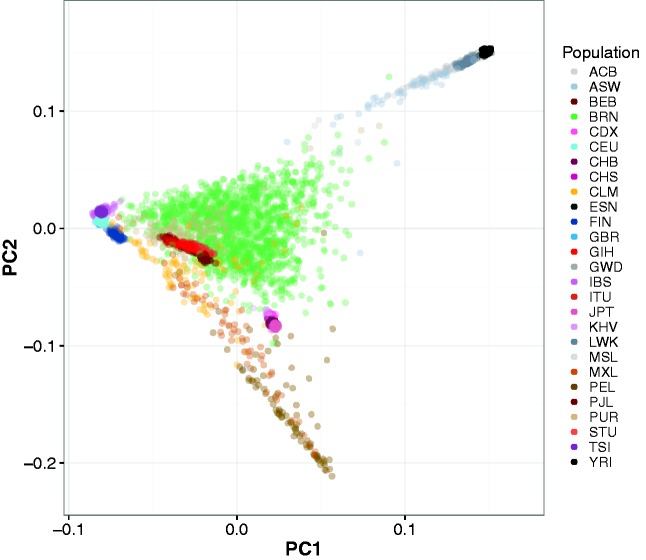
Genome-wide principal component analysis of the Brazil samples using all 1000 Genomes as race/ethnic reference samples in the component coordinates. The principal components were solely defined by variation in the Brazil samples (shown in green glyphs, BRN group) and the reference samples plotted into these coordinates. Principal Component 1 (PC1) is plotted against 2 (PC2). The 1000 Genomes population descriptions corresponding to the three letter codes are listed in supplementary table S2, Supplementary Material online. Higher principal components (PC3–15) did not define additional ancestral structure at the granularity of the 1000 Genomes populations. European populations cluster at approx. (PC1, PC2) (−0.08, 0.01), Asian populations at (0.02, −0.08) and Indian subcontinent populations at (−0.03, −0.02). African populations and Amerindian populations cluster along the two axes projecting from Europe at the top and bottom of the plot.

Figure 2 shows the results from the initial unsupervised principal component analysis (PCA) of the 1,538 Brazil samples with all publicly available 1000 Genomes (1KG) population samples in the 2013 release (1000 Genomes Project Consortium et al. 2012) projected onto the first two axes of maximal genetic variation defined solely by the Brazil samples (PC1 × PC2). The full 1KG populations and three letter abbreviations are explained in supplementary table S2, Supplementary Material online and subsets in tables 2 and 3. Brazilians from North-Eastern Brazil, labeled BRN, are plotted in green glyphs throughout. Using only the BRN samples to define the principal components (PCs) identifies and ranks the predominant axes of variation, compared to projection onto all samples which addresses the different question of the amount of each ancestry within the samples defined by the proxy populations. Projection of the 1KG samples onto the BRN axes identified a triangular structure of three continental clusters with PC1 as a predominantly Africa—Europe admixture axis and PC2 separating Native American genetic variation from the European and African poles. Hence the Brazil samples contained African, European, and Native American ancestry and admixture between these continental groups explained the greatest fraction of the genetic variation in the samples. At the negative Native American pole of the PC2 axis the density of Lima Peruvian 1KG samples (PEL) increased, with outlier samples from other Hispanic 1KG populations including Mexicans from Los Angeles (MXL) and Columbians from Medellin (CLM). This suggested that the PEL group might form the basis of a latter day proxy for the Native American ancestry component within the Brazilians. Higher principal components 3–15 defined latent variation within the Brazil samples without additional structure in the 1KG reference samples. For comparison purposes, we generated PCA plots using all BRN and 1KG samples in a joint analysis of total genetic variation; these are available in supplementary figures S15–S28, Supplementary Material online, following other supplementary figures.

**Table 2.** Genetic Differentiation (Fst) between the Ancestral Brazilian Admixture Components from an Unsupervised ADMIXTURE Analysis and Reference 1000 Genomes Populations Containing Major African, Latin American, and European Ancestry

1KG Code^a^	Population^a^	BRN1 (Afr)^b^	BRN2 (Amr)^b^	BRN3 (Eur)^b^
BRN^c^	Brazilians in North-East Brazil	0.0256 (254, 258)	0.0192 (191, 193)	0.0108 (108, 109)
Africa				
ASW	African Ancestry in Southwest US	0.0086 (086, 088)	0.0705	0.0727
ACB	African Caribbean in Barbados	0.0157 (156, 158)	0.0908	0.0954
LWK	Luhya in Webuye, Kenya	0.0240 (238, 242)	–	–
YRI	Yoruba in Ibadan	0.0269 (267, 271)	–	–
ESN	Esan in Nigeria	0.0278 (276, 281)	–	–
GWD	Gambian in Western Division	0.0281 (279, 283)	–	–
MSL	Mende in Sierra Leone	0.0286 (284, 288)	–	–
Latin America				
MXL	Mexican Ancestry in Los Angeles	0.0632	0.0123 (122, 125)	0.0297
CLM	Colombian in Medellin	0.0482	0.0201 (200, 203)	0.0110
PEL	Peruvian in Lima	0.0983	0.0220 (218, 222)	0.0754
PUR	Puerto Rican in Puerto Rico	0.0396	0.0286 (285, 289)	0.0066
Europe				
IBS	Iberian in Spain	0.0631	0.0529	0.0032 (032, 033)
TSI	Toscani in Italy	0.0643	0.0538	0.0041 (041, 043)
CEU	North/Western European ancestry in Utah	0.0667	0.0527	0.0050 (050, 051)
GBR	British in England and Scotland	0.0672	0.0532	0.0053 (052, 054)
FIN	Finnish in Finland	0.0701	0.0518	0.0118 (118, 120)

a 1KG populations BEB, CDX, CHB, CHS, GIH, ITU, PJL, JPT, KHV, and STU are not shown since they were not in the closest population groups by ranked Fst and were not predominantly of the putative ancestral group.

b The 95% confidence interval (CI) from bootstrap percentile (B = 10,000 replicates) is shown for the closest 1KG populations for each inferred component ancestry as last 3 digits only. CIs are not shown for other Fst values. – (dash) indicates Fst >0.1 (not shown for clarity).

c BRN row shows the Fst with the source Brazil samples without segregation of putative ancestral components for comparison.

**Table 3.** Estimated Genetic Differentiation (Fst) between the Brazil Ancestral Admixture Components from a Supervised ADMIXTURE Analysis and Reference 1000 Genomes Populations Containing Major African, Latin American, and European Ancestry

1KG Code^a^	Population^a^	BRN1 (Afr)^b^	BRN2 (Amr)^b^	BRN3 (Eur)^b^
BRN^c^	Brazilians in North-East Brazil	0.0837 (832, 843)	0.0801 (796, 806)	0.0154 (153, 155)
Africa				
ASW	African Ancestry in Southwest US	0.0132 (131, 134)	–	0.0821
ACB	African Caribbean in Barbados	0.0074 (073, 075)	–	–
LWK	Luhya in Webuye, Kenya	0.0102 (101, 103)	–	–
YRI	Yoruba in Ibadan	0.0040 (039, 040)	–	–
ESN	Esan in Nigeria	0.0036 (035, 036)	–	–
GWD	Gambian in Western Division	0.0107 (106, 108)	–	–
MSL	Mende in Sierra Leone	0.0084 (084, 085)	–	–
Latin America				
MXL	Mexican Ancestry in Los Angeles	–	0.0375 (372, 377)	0.0362
CLM	Colombian in Medellin	–	0.0689 (684, 693)	0.0148
PEL	Peruvian in Lima	–	0.0076 (075, 077)	0.0867
PUR	Puerto Rican in Puerto Rico	–	0.0911 (905, 917)	0.0094
Europe				
IBS	Iberian in Spain	–	–	0.0013 (012, 013)
TSI	Toscani in Italy	–	–	0.0030 (029, 031)
CEU	North/Western European ancestry in Utah	–	–	0.0039 (038, 040)
GBR	British in England and Scotland	–	–	0.0041 (040, 042)
FIN	Finnish in Finland	–	–	0.0117 (115, 118)

a 1KG populations BEB, CDX, CHB, CHS, GIH, ITU, PJL, JPT, KHV, STU are not shown since they were not in the closest population groups by ranked Fst and were not predominantly of the putative ancestral group.

b Analogous to table 2, the supervised ADMIXTURE analysis of the Brazil samples used *N* = 30 reference sample genome-wide profiles from each of the ancestral proxy groups. The 95% confidence interval from bootstrap percentile (10,000 replicates) is shown for the closest 1KG populations for each inferred component ancestry as last 3 digits only. – (dash) indicates Fst > 0.1 (not shown for clarity). CIs are not shown for other Fst values.

c BRN row shows the Fst with the source Brazil samples without segregation of putative ancestral components.

### Unsupervised Admixture Analysis on Brazilian Samples Recapitulated the PCA Results to Identify Major Admixing Ancestral Groups

We recapitulated the PCA results in an unsupervised analysis using ADMIXTURE 1.23 (Alexander et al. 2009; Alexander and Lange 2011). We tested all models from *K* = 1–10 for the Brazil samples and found that *K* = 3 minimized the cross-validation error, suggesting a latent three ancestral cluster model best fitted the data (supplementary fig. S1, Supplementary Material online). We computed pairwise Hudson Fst values (Bhatia et al. 2013) between the three inferred ancestral components and 1KG populations (table 2). The first ancestral component in the North-Eastern Brazil samples (BRN1) was genetically closest to 1KG populations containing a significant African component, but was closest to African Americans in the US South-West (ASW) and African Caribbeans in Barbados (ACB), populations containing European admixture. This was most likely due to imperfect partitioning of genetic variance between admixing continental components and European admixture retained within the inferred Brazil African component. Of the 1KG populations recruited in Africa, the Luhya in Webuye, Kenya (LWK) were more genetically similar by Fst than the Western sub-Saharan African populations of Yoruba in Nigeria (YRI), Esan in Nigeria (ESN), Gambians in Western Division (GWD), and Mende in Sierra Leone (MSL). The third component, BRN3, was closest to European populations, notably the Southern European populations from Spain (IBS) and Italy (TSI). The second component BRN2 was closest to Latin American recruited populations, Mexicans in Los Angeles (MXL), Columbians in Medellin (CLM), and then PEL, indicating a major Native American component. The population ranking by similarity again reflected imperfect partitioning and residual nonAmerindian admixture.

### Supervised Africa-Centric PCA Identified Recent West-Central African Admixture in the Brazilians and More Ancient Similarity to Ancestral East African Populations

**Fig. 3.**
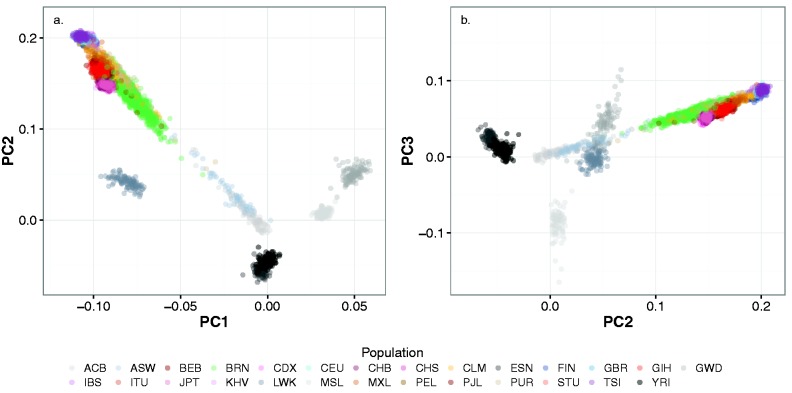
Genome-wide, Africa-centric, principal component analysis of the Brazil and 1000 Genomes samples. The sample three letter and color codes are identical to figure 1. Genetic variation within the African 1000 Genomes populations (ESN, GWD, LWK, MSL, and YRI), defined (supervised) the principal components with other samples plotted onto these coordinates. Panel a shows PC1 vs. PC2 and panel b shows PC2 vs. PC3. Principal components higher than three reflected recent kinship within a 1000 Genomes African population, rather than ancestral population structure. Approximate coordinates of the supervising 1000 Genomes African clusters are (PC1, PC2): YRI/ESN (0.0, −0.05); MSL (0.03, 0.001); GWD (0.05, 0.05); LWK −0.075, 0.04). Nonsupervising samples with majority African ancestry include the more “smeared” (admixed) and less homogeneous ACB (−0.02, 0.0); and ASW (−0.02, 0.02). PC3 segregates the MSL population (−ve PC3 coordinate).

To better understand the finding that the African (BRN1) ancestral admixture component was most similar to the LWK population, we performed a supervised Africa-centric PCA and projected the remaining 1KG and Brazil samples on to genetic coordinates generated from the 1KG samples of sub-Saharan populations in Africa (ESN, YRI, GWD, MSL, and LWK; fig. 3). PC1 distinguishes an East-West sub-Saharan axis with the Luhya from Kenya (LWK) samples at most negative PC1 to The Gambian (GWD) samples most positive, with the Yoruban (YRI) and Esan (ESN) Nigerian populations intermediate. The Brazil (BRN) and remaining 1KG populations clearly segregated with the Central African LWK samples on this axis, revealing the African source of ancient founding populations of Europe, Asia, and the Americas (Bryc et al. 2010). PC2 in figure 3 distinguishes the degree of Eurasian admixture in the sub-Saharan African populations, with more Eurasian admixture in The Gambia (GWD) and Kenya (LWK) than in the Mende of Sierra Leone (MSL) or YRI/ESN (Bhatia et al. 2011; Gurdasani et al. 2015). The Brazilians (and ACB and ASW) are smeared along an axis from the centroid of the nonAfrican 1KG cluster towards the West-Central African YRI/ESN, representing more recent admixture with African populations that derived from near modern day Nigeria and the Gulf of Guinea, rather than further West towards Sierra Leone, Senegal, and Gambia. PC3 segregated the Mende MSL from the other West and Central African populations.

### Proxy Population Samples for the Amerindian and African Components of Ancestry in North-Eastern Brazil

The admixture estimates from an unsupervised admixture analysis are known to be potentially biased and inaccurate (Tang et al. 2005; McVean 2009) so including samples of the source admixing populations in a supervised admixture analysis would likely yield more accurate estimates (Alexander and Lange 2011). The analysis above suggested the Iberian Spanish (IBS) samples as a proxy for the European but in the absence of a large enough sample size of known, nonadmixed native Amerindian samples available as a proxy, we sought to develop an ancestral proxy for the native Amerindian ancestry using available public genome-wide genotype data. We ranked the 1000 Genomes reference samples by their increasing PC2 coordinate in figure 2 and selected Amerindian proxy samples from the samples with the most negative PC2 values, that is, containing the smallest proportion of nonAmerindian admixture, up to a maximum of N(proxy) total samples. As seen in figure 2, the most extreme PC2 samples are predominantly Peruvians from Lima (PEL) with smaller numbers of Mexicans (MXL) and Columbians (CLM). We used a similar strategy to select African proxy samples. Although the 1KG LWK African population from Africa had the highest overall genetic similarity to the Brazil African ancestral component, the goal of the admixture analysis was to remove recent admixture and therefore YRI/ESN were predominantly selected as African component supervising proxy samples from the most extreme positive PC1. The numbers of samples from each 1000 Genomes group as a function of total N(proxy) are shown in supplementary table S3, Supplementary Material online.

### Estimates of Admixture Proportions in the Brazil Samples Using Supervised Admixture Analysis

**Fig. 4.**
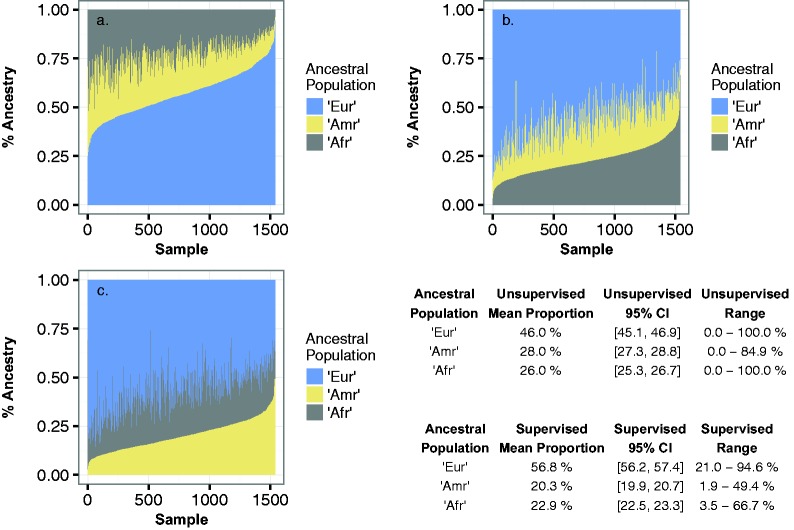
The proportion of continental ancestry within the Brazil samples, estimated using supervised ADMIXTURE analysis. The *K* = 3 ancestral components are labeled “Eur” predominantly European; “Amr” predominantly Amerindian; “Afr” predominantly African. In each panel, each individual sample along the *x*-axis is a narrow vertical bar with three color intervals along the *y*-axis that are proportional to the percentage of the three ancestries and sum to 100% (*y* = 1.0). In panel a, the Brazil samples are sorted along the *x*-axis from lowest to highest fraction of Eur ancestry (blue); in panel b, sorted by fraction of African ancestry (grey); in panel c, by fraction of Amerindian ancestry (yellow). The table shows the mean proportion of each ancestry with 95% confidence interval, and range.

Having established the best 1KG proxy samples, we identified the optimal number of proxy samples to use for the supervised analysis to maximize the precision and minimize the bias of the estimates of ancestral proportions. We varied the equal number of ancestral samples from each proxy group in supervised ADMIXTURE analyses with bootstrap estimates of standard error of ancestry proportions, reasoning that the standard error and precision would decrease with the addition of more proxy samples until a minimum was reached after which addition of more samples would increase the standard error. In supplementary figure S2, Supplementary Material online, we show that a minimum was reached with *N* = 30 samples of each of the three ancestral proxy groups although the minimum is broad and little changed with *N* = 20–50, so that even a relatively small number of supervising samples can be valuable in reducing variance of the estimates. We used *N* = 30 samples for all other supervised analyses of the Brazil samples in this work. The resulting changes in estimated ancestral proportions are shown in figure 4 and the improved Fst estimates are shown in table 3. We labeled the three ancestral components as European (Eur), Amerindian (Amr), and African (Afr), based on similarity to the contemporary proxy populations. The mean proportion of European ancestry increased from 46.0% to 56.8% with attendant decreases in the mean proportion of Amerindian and African ancestry. Supplemental table S4, Supplementary Material online shows the estimates of mean ancestry in each of the six study groups of table 1. There was no significant difference in the mean ancestry between any pair of the five study groups located in Fortaleza after multiple testing correction (all *P*-value > 0.05/15), but the mean ancestry in the Recodisa case control group (enrolled in six other cities across four North-Eastern states) differed from all five Fortaleza groups (*P* < 1 × 10^−10^). The participants in Recodisa had slightly higher mean European (61% vs. 52–55%) and African ancestry (24% vs. 21–22%) and were more variable in these components, while their mean Amerindian ancestry was lower (15% vs. 24–26%) and less variable.

### The Most Highly Genetically Differentiated Loci along the Amerindian Branch from an Ancestral Population of the Three Admixture Components of Brazil

**Fig. 5.**
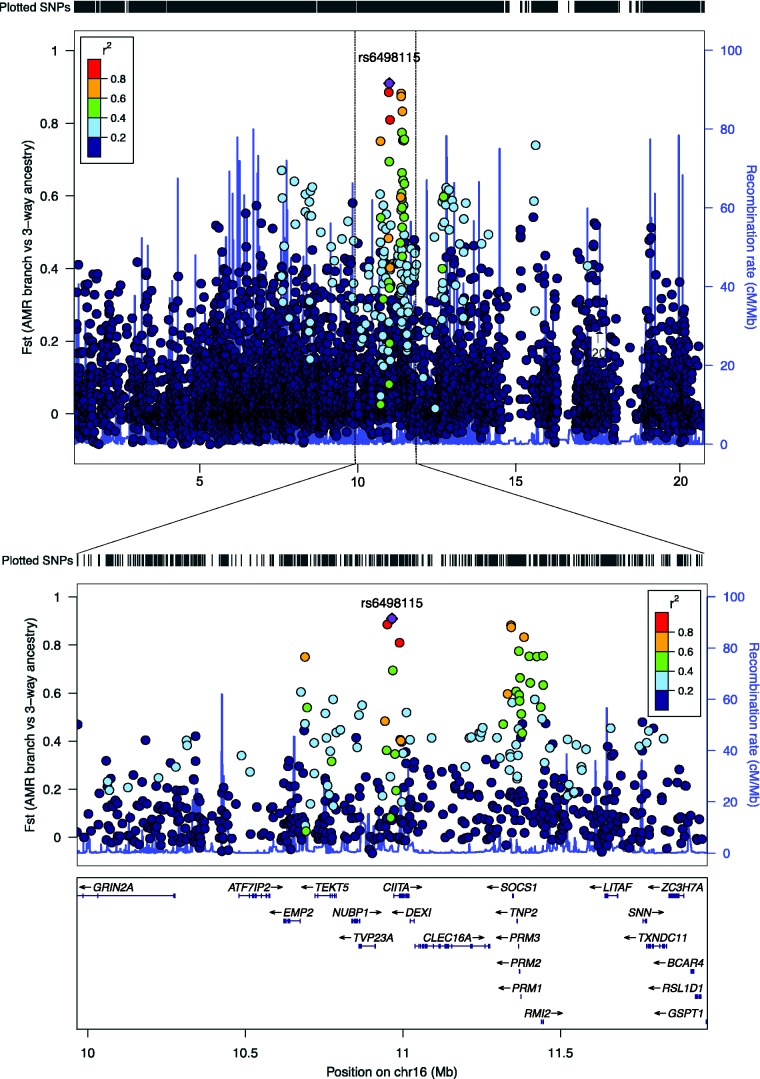
Genome regional plots of the most highly differentiated region along the Amerindian branch, centered on SNP rs6498115, chromosome 16. The plots were generated using LocusZoom and show the physical region of chromosome 16, 0.9–20.9 Mb and at higher resolution, 9.9–11.9 Mb. The linkage disequilibrium between SNPs was estimated in LocusZoom using the 1000 genomes admixed American samples (AMR).

We used the method of locus-specific branch length (Shriver et al. 2004; Mattiangeli et al. 2006; Bigham et al. 2010; Bhatia et al. 2011) to compute the Fst statistic for the latter day Amerindian ancestry component in the Brazil samples relative to a hypothetical single ancestral population from which the European, Amerindian, and African admixture components emerged. This is monotonically related to the population branch statistic method as described in Materials and Methods. The overall distribution of the 400,150 SNP Fst values was exponential-like in the right hand tail, with mean, median, and 75% percentile Fst values of 0.079, 0.041, and 0.126, respectively. Table 4 shows the top 10 SNPs ranked by greatest locus branch-specific Fst values which segregate into five distinct regions. The top differentiated SNP (rs6498115) was located within the proximal promoter of the Class II, Major Histocompatibility Complex, Transactivator (*CIITA)* gene, a positive regulator of class II major histocompatibility complex gene transcription, supplementary figure S3, Supplementary Material online, with two additional differentiated flanking SNPs in linkage disequilibrium with the top SNP (*r*^2^ > 0.8; fig. 5). Other more distal SNPs with reduced Fst at a lower linkage disequilibrium threshold (*r*^2 ^> ^ ^0.6) extend approximately 500 kb 5′ and 3′ to the leading SNP, extending the physical gene loci covered to *EMP2* (5′) and the *PRM1*/*RMI2* gene cluster (3′). SNP rs6498115 lies within prominent H3Kme1 and H3K27Ac marks defined by seven ENCODE cell lines (http://genome.ucsc.edu; last accessed October 16, 2016) and in four of the seven, these marks lie in regions predicted to have strong enhancement (53/125 cell lines also demonstrate DNase hypersensitivity). As shown in supplementary figure S3, Supplementary Material online, two SNPs in the NHGRI genome-wide association study catalog fall within this interval (Welter et al. 2014), rs4781011 within the *CIITA* gene, from a secondary analysis in a case–control association study of ulcerative colitis in Europeans (McGovern et al. 2010), and rs6498142 within the neighboring *CLEC16A* gene, from a case–control association analysis of acute coronary syndrome in Mexican Americans (Vargas-Alarcon et al. 2014). The other SNPs in table 4 within the *CIITA* linkage disequilibrium region were rs35346036 (within 100 kb of *CIITA*); rs45601437 (*CIITA* intron); rs77979769 (within 10 kb 3′ of *SOCS1*); rs2021760 (within 10 kb 3′ of *SOCS1*); rs8054781 (within 10 kb 3′ of *PRM1*). The other four regions in table 4 were: rs1834619 (*SMC6* gene intron), chromosome 2 at 17.9 Megabases (Mb), shown in supplementary figure S4, Supplementary Material online; rs288697 (*KLHL29* gene intron), chromosome 2 at 23.9 Mb, supplementary figure S5, Supplementary Material online; rs2866065 in an extended gene desert region on chromosome 16 at 75.8 Mb, supplementary figure S6, Supplementary Material online; and rs16964480 (*MEIS2* gene intron), chromosome 15 at 37.3 Mb, supplementary figure S7, Supplementary Material online.

**Table 4.** The Top 10 Most Highly Differentiated Loci for the Amerindian Admixture Branch within the Brazil Samples Compared with the Putative Ancestral Population of the Three Admixture Components

CHR	SNP	GENPOS^a^	POS^a^	A1^a^	A2^a^	f(Eur)^b^	f(Amr)^b^	f(Afr)^b^	Fst^c^	Nearest Gene^d^
16	rs6498115	28.170	10965511	T	C	0.000	0.908	0.000	0.908	*CIITA*, promoter
2	rs1834619	39.407	17901485	A	G	0.041	0.942	0.000	0.899	*SMC6*, intron
16	rs77979769	28.358	11343560	A	G	0.073	0.949	0.035	0.883	*SOCS1*, 3’ downstream
2	rs2288697	47.776	23860168	A	G	0.029	0.906	0.018	0.877	*KLHL29*, intron
16	rs35346036	28.164	10951098	G	A	0.065	0.957	0.088	0.872	CIITA
16	rs2021760	28.358	11343992	G	A	0.076	0.947	0.065	0.869	*SOCS1*, 3’ downstream
16	rs45601437	28.180	10989754	A	G	0.006	0.912	0.050	0.862	*CIITA*, intron
16	rs2866065	91.637	75822042	A	G	0.075	0.929	0.000	0.849	–
16	rs8054781	28.400	11384776	C	T	0.026	0.932	0.092	0.846	*PRM1*, promoter
15	rs16964480	35.881	37284909	G	T	0.000	0.837	0.000	0.837	*MEIS2*, intron

a GENPOS is the genetic map position of the marker on a chromosome (CHR) in centiMorgans, POS is the hg19 physical map position, A1 is the reference allele, A2 the alternative.

b f(Afr), f(Amr), f(Eur) are the estimated reference allele frequencies for SNP A1 allele in the 3 Brazil ancestral admixture components.

c This table shows the top ten ranked loci by Hudson Fst value, where Fst measures the genetic differentiation between the inferred second Amerindian admixture component (Amr) and a single ancestral population of all components. Fst is the Amerindian component branch-specific estimate of genetic differentiation.

d Nearest Gene is taken from the RefSeq track in the UCSC genome browser database (http://genome.ucsc.edu; last accessed October 16, 2016). Annotated SNPs are within 100 kb of the nearest gene. A promoter SNP is within 10 kb 5′ to the transcription start site; 3′ downstream SNP is within 10 kb 3′ of the nearest gene; exon and intron are within an exon or intron of the nearest gene.

### The Most Highly Differentiated Loci for Amerindian Ancestry in Brazil Compared with the Closest Population in 1000 Genomes Asian Populations

We were interested in identifying SNPs that were most highly differentiated in the Amerindian ancestral component compared with the closest available Asian population, conjecturing that the most highly differentiated SNPs might be candidates for disease or trait loci under ancestral selection during the migration from Asia into the Americas, and could differentiate disease risk in Amerindians from ancestral Asian populations. Comparison of these SNPs to the Amerindian differentiated SNPs identified above could suggest whether the differentiation was more likely to have occurred during the Asia to Americas ancestral migration. We estimated the population Fst values for the Amerindian admixture component in Brazil, BRN2 (Amr), versus all Asian 1KG populations that were recruited from within Asia as shown in supplementary table S5, Supplementary Material online. Surprisingly, Bengalis in Bangladesh (BEB) were significantly closer to the BRN2 (Amr) admixture component than any of the other 1KG populations as measured by Fst, with the Punjabis from Lahore (PJL) the next closest in the second cluster. We ran TREEMIX 1.12 to better understand the apparent relationship between BEB and the BRN2(Amr) component (Pickrell and Pritchard 2012). We included BRN2(Amr), all Asian 1KG populations and YRI as an outgroup and varied the number of migrations from 0 to 8. Since the 5-migration model was only slightly worse than a 6-migration model based on the change in log likelihood and a distinct flattening in the log likelihood change profile (supplementary fig. S8, Supplementary Material online), and the 5-migration model generated acceptable residuals (supplementary fig. S9, Supplementary Material online), we accepted this is as the preferred model. The phylogenetic plot (supplementary fig. S10) illuminates the source of the similarity in BEB and shows that the Amerindian component is most genetically similar to an admixed Central-East Asian ancestral group containing later admixture between a descendent group of the North-Indian subcontinent clade (PJL, GIH, and BEB) and an older South-East Asian/Japan lineage, albeit with evidence of later reverse gene flow between the Indian and East Asian clades.

Similar to the LSBL test, the distribution of the Fst values was also exponential-like with mean, median, and 75% percentile Fst values of 0.083, 0.045, and 0.123 respectively.

The top 10 most highly differentiated loci by Fst between the BRN2(Amr) component and the BEB population are shown in table 5, were located in eight genome regions. The top SNP (rs7631391) is present in an intron of the gene ADAM metallopeptidase with thrombospondin type 1 motif, 9, (*ADAMTS9)* located on chromosome 3 at 64.5 Mb, supplementary fig. S11, Supplementary Material online. This SNP was also within the top 500 SNPs from the previous Amerindian locus specific branch analysis (first green track in supplementary fig. S11, Supplementary Material online). The other SNPs were located in seven other regions: rs77594147 and rs73318286 within *DOCK2* gene introns, chromosome 5 at 169.2 Mb (supplementary fig. S12, Supplementary Material online); rs28649017 in *SLC28A1*, chromosome 15 at 85.4 Mb (supplementary fig. S13, Supplementary Material online); rs71519991 5 kb 3′ to the gene *ARHGAP5*, chromosome 14 at 32.6 Mb (supplementary fig. S14, Supplementary Material online); rs45601437 and rs6498115 in the *CIITA* gene intron and promoter, chromosome 16 at 11.0 Mb (supplementary fig. S3, Supplementary Material online); and rs6088519 (*MAP1LC3A* gene promoter, chromosome 20, 33.1 Mb), rs4666032 (*BRE* gene intron, chromosome 2, 28.3 Mb), and rs117487308 (*SLC25A17* gene exon, chromosome 22, 41.2 Mb). The genome interval annotation plots for *CIITA*, *ADAMTS9*, *DOCK2*, and *ARHGAP5* displaying the most differentiated SNPs for both the Fst tests of ancestral locus branch length and differentiation from the Asian BEB population contain highly differentiated SNPs in both tracks.

**Table 5.** The Top 10 Most Highly Differentiated Loci for the Amerindian Admixture Component in the Brazil Samples vs. the Closest Asian 1KG Population (BEB, Bengalis in Bangladesh)

CHR	SNP	GENPOS	POS	A1	A2	f(Eur)	f(Amr)	f(Afr)	f(BEB)^a^	Fst^b^	Nearest Gene^c^
3	rs7631391	88.546	64514393	G	A	0.002	0.950	0.113	0.058	0.885	*ADAMTS9*, intron
5	rs77594147	178.800	169155975	G	A	0.079	0.878	0.328	0.017	0.857	*DOCK2*, intron
5	rs73318286	178.809	169162708	G	A	0.042	0.879	0.297	0.029	0.843	*DOCK2*, intron
15	rs28649017	89.197	85438991	A	G	0.353	0.150	0.515	0.983	0.827	*SLC28A1*, exon/intron
14	rs7151991	30.033	32635572	A	G	0.148	0.950	0.158	0.116	0.821	*ARHGAP5*, 3’ downstream
16	rs45601437	28.180	10989754	A	G	0.006	0.912	0.050	0.081	0.816	*CIITA*, intron
16	rs6498115	28.170	10965511	T	C	0.000	0.908	0.000	0.081	0.811	*CIITA*, promoter
20	rs6088519	57.089	33132191	T	C	0.298	0.966	0.249	0.163	0.791	*MAP1LC3*, promoter
2	rs4666032	50.639	28254769	C	T	0.000	0.827	0.000	0.029	0.788	*BRE*, intron
22	rs117487309	49.210	41195082	A	G	0.039	0.786	0.000	0.000	0.786	SLC25A17, exon

a All other columns are as in table 4, except f(BEB) contains the allele frequency of the A1 reference allele estimated in *N* = 86 Bengalis in Bangladesh 1KG samples.

b This table shows the top ten ranked loci by Hudson Fst value, where Fst measures the genetic differentiation between the Amr Amerindian admixture component and the BEB Bangladesh 1KG population.

c Nearest Gene is taken from the RefSeq track in the UCSC genome browser database (http://genome.ucsc.edu; last accessed October 16, 2016). Annotated SNPs are within 100 kb of the nearest gene. A promoter SNP is within 10 kb 5′ to the transcription start site; 3′ downstream SNP is within 10 kb 3′ of the nearest gene; exon and intron are within an exon or intron of the nearest gene. SNP rs28649017 in *SLC28A1* is in an exon and intron of different splice forms of the gene transcript.

To better understand the genetic history of the top differentiated SNPs and test for the possibility that the differentiation arose from founder effects within Asia, we computed the allele frequencies and locus-specific Fst values for ten of the 1KG populations: seven Asian populations resident within Asia countries; and Gujarati from Houston (GIH), LWK (Kenyans from Webuye), and TSI (Italians from Tuscany) as proxies for the ancestral African and European populations (table 6). TSI was chosen as the geographically closest population to the Levantine migration routes out of Africa although IBS (Spaniards) gave very similar results. In the five SNPs, we found three distinct patterns in the Fst and allele frequencies across the populations. SNPs in *ADAMTS9* (rs7631391, chr3), *ARHGAP5* (rs71519991, chr14), and *CIITA* (rs45601437, chr16) showed a trend from high Fst, low frequency in Africa with incremental Fst decreases and allele frequency increases in Asian populations, with the largest change occurring in the BRN2(Amr) component. The *DOCK2* region (rs77594147, chr5) showed a low Fst in Africa (0.541 allele frequency) but higher Fst and frequency <0.10 in all other populations except Brazil Amerindian. The third pattern in *SLC28A1* (rs28649017, chr15) was an increasing Fst and increasing allele frequency from Africa to BEB and PJL and then a dramatic decrease in Fst and allele frequency in the rest of Asia and BRN2(Amr).

**Table 6.** Locus-Specific Fst and Allele Frequencies within the 1000 Genomes Populations for the Five Most Differentiated SNPs in Distinct Genome Regions in the Brazil Amerindian Component vs. Closest Asian Population (BEB)

Population^a^			LWK	TSI	BEB	PJL	CHB	JPT	CHS	GIH	KHV	CDX	BRN
N^b^			97	107	86	96	103	104	105	101	99	93	1538
SNP CHR:POS	A1	A2	Fst LWK^c^	Fst TSI^c^	Fst BEB^c^	Fst PJL^c^	Fst CHB^c^	Fst JPT^c^	Fst CHS^c^	Fst GIH^c^	Fst KHV^c^	Fst CDX^c^	
rs7631391 3:64514393	G	A	0.858	0.950	0.885	0.909	0.634	0.685	0.682	0.889	0.671	0.694	
rs77594147 5:169155975	G	A	0.240	0.826	0.857	0.801	0.782	0.777	0.795	0.817	0.822	0.785	
rs28649017 15:85438991	A	G	0.102	0.242	0.827	0.843	−0.003	0.024	0.052	−0.001	0.008	−0.003	
rs7151991 14:32635572	A	G	0.813	0.754	0.821	0.749	0.794	0.791	0.766	0.677	0.749	0.707	
rs45601437 16:10989754	A	G	0.827	0.901	0.816	0.820	0.511	0.586	0.567	0.865	0.628	0.629	

SNP CHR:POS	A1	A2	f(A1) LWK^d^	f(A1) TSI^d^	f(A1) BEB^d^	f(A1) PJL^d^	f(A1) CHB^d^	f(A1) JPT^d^	f(A1) CHS^d^	f(A1) GIH^d^	f(A1) KHV^d^	f(A1) CDX^d^	f(A1) BRN2Amr^d^
rs7631391 3:64514393	G	A	0.082	0.000	0.058	0.036	0.286	0.240	0.243	0.054	0.253	0.231	0.950
rs77594147 5:169155975	G	A	0.541	0.042	0.017	0.063	0.078	0.082	0.067	0.050	0.045	0.075	0.878
rs28649017 15:85438991	A	G	0.356	0.500	0.983	0.995	0.141	0.245	0.291	0.173	0.101	0.145	0.150
rs7151991 14:32635572	A	G	0.124	0.178	0.116	0.182	0.141	0.144	0.167	0.248	0.182	0.220	0.950
rs45601437 16:10989754	A	G	0.072	0.009	0.081	0.078	0.345	0.279	0.295	0.040	0.242	0.242	0.912

a The populations are ordered by Africa(LWK), Europe(TSI), Asia(BEB, PJL, CHB, JPT, CHS, GIH, KHV, and CDX) where Asian populations are in decreasing order of similarity to BRN2(Amr) (supplementary table S5, Supplementary Material online). Population codes are LWK (Luhya in Webuye, Kenya); TSI (Toscani in Italy); BEB (Bengalis in Bangladesh); PJL (Punjabis in Lahore, Pakistan); CHB (Han Chinese in Beijing); JPT (Japanese in Tokyo, Japan); CHS (Southern Han Chinese); GIH (Gujarati in Houston, USA); KHV (Kinh in Ho Chi Minh City, Vietnam); CDX (Chinese Dai in Xishuangbanna); BRN2(Amr) (North-Eastern Brazilians, Amerindian admixture component 2).

b Number of DNA samples within each population.

c SNP-specific Fst value for each population compared with the Brazil Amerindian admixture component, BRN2 (Amr). In the top half of the table, the BEB Fst values are shown in bold since this is the closest Asian population and are the values in table 5. In the lower half of the table, the BEB and BRN2(Amr) columns are in bold since the difference in these frequencies is used in the FstBEB calculation. Other Fst 1KG values in the top half of the table are calculated from the 1KG population and BRN2(Amr) frequencies in the lower half.

d Frequency of the A1 allele in each population.

## Discussion

Brazil poses a complex methodological problem for genetic analysis due to extensive recent admixture between individuals of European, African, and Native American Indian descent, combined with a complex history of migration and forced slavery. This complexity is an advantage for disease gene mapping because it allows the interrogation of wider genetic variation and resulting clinical and biological effects. Our purpose in this study was to develop accurate estimates of ancestry from genome-wide SNP data and use the jointly fitted SNP allele frequencies in a genome-wide scan for the most highly differentiated loci in the Amerindian ancestry component. Although not proven, these loci are strong candidates for having been under selection pressure. We chose to focus on Amerindian locus differentiation within our Brazil population since less work has been possible on Native Amerindian population genetics due to community sensitivities and fewer publicly available large Amerindian genome-wide data sets.

By ranking SNPs in the upper tail of the genome-wide distribution of Fst values from a locus-specific branch length (LSBL) test of reconstructed Amerindian ancestry in Brazilians, we found five genome regions containing the top 10 SNPs which were the most differentiated SNPs, and therefore candidates for selection (table 4 and fig. 5). The LSBL analysis against the single ancestral root population is a model for testing SNP locus differentiation but is not intended to be a literal model of the archaic history of the continental populations and does not model complex ancestral history, replacement, or migrations between Africa, Europe, and Americas. The top differentiated SNP (rs6498115) was located within the proximal promoter of the *CIITA* gene, 6 kb (kb) upstream of the start site of *CIITA* transcription, within prominent H3Kme1 and H3K27Ac epigenetic marks in a region of transcriptional enhancement, and is a strong positional and functional candidate as transcriptional regulator of *CIITA* expression. The LD region for this SNP extended from *EMP2* (5′) to the *PRM1*/*RMI2* gene cluster (3′). The second SNP was in an intron of the structural maintenance of chromosomes 6 gene *SMC6* (rs1834619), a gene that is obligate for normal development and chromosome structure but without prior clinical research results or GWAS signals to suggest possible beneficial changes in associated human phenotypes. Other SNPs were located in an intron of kelch-like family member 29 (*KLHL29*) rs2288697, a gene desert region of chromosome 16 (rs2866065, 75.8 Mb) approx. 5 kb from a localized isolated genome region conserved in mammals and with a cluster of transcription factor sites identified by CHIP-Seq experiments, (http://genome.ucsc.edu; last accessed October 16, 2016), and in an intron of Meis homeobox 2 (*MEIS2)* rs16964480.

In a second similar analysis of pairwise Fst values computed between the Brazilian Amerindian admixture component and the closest Asian population, Bengalis in Bangladesh (BEB), we found evidence of extreme differentiation of the top 10 SNPs in eight regions in the reconstructed Amerindian branch compared with a single ancestral root population (table 5). By comparing these two analyses we hoped to gain insight into where and when in the complex history of the Amerindian population, the locus-specific differentiation may have occurred, pre- or during Asia-to-Americas migration. The only region that contained SNPs ranking in the top 10 SNPs of both analyses was the *CIITA* region, and as table 6 showed, the greatest component of the differentiation is most likely to have occurred in the geographical migration between Asia and ending in the North-Eastern region of Brazil, although may already have begun in Asia. The LSBL test region *SMC6* (rs75594147, chromosome 2) also contained SNPs within the top 500 of the Amerindian vs. Asia analysis (second green track, supplementary fig. S4, Supplementary Material online), which provides suggestive evidence for differentiation during the peopling of the Americas. We also found four other interesting regions in the Amerindian vs. Asian analysis, but which did not appear in the top 10 LSBL SNPs, although the *ADAMTS9* (rs7631391, chromosome 3), *DOCK2* (rs77594147, chromosome 5), and *ARHGAP5* (rs7151991, chromosome 14) regions also contained SNPs that were ranked in the top 500 of the LSBL test (top green track, supplementary figs. S11–S13, Supplementary Material online, respectively). From comparison of the pairwise Fst and allele frequencies across the 10 populations with the Brazil Amerindian in table 6, *ADAMTS9* and *ARHGAP5* showed a smooth trend of decreasing Fst and increasing allele frequency from Africa to Asia with the largest change in Fst in the transition to Amerindian also suggesting these are good candidates for New World selection. Of the other top gene regions, *DOCK2*, showed a much higher allele frequency in Africa than in the European or Asian populations, and higher still in Brazil Amerindian. One possible explanation is that gene variant was originally at low frequency in the founding African migration, but experienced selection pressure separately and independently in Africa and the Americas. This pattern explains why the LSBL test for the SNP did not yield an extreme Fst statistic yet was highly differentiated in the Asian comparison. The final gene region, *SLC28A1* (rs28649017, chromosome 15) contains a SNP that rose in Fst and allele frequency from Africa to Europe to Bangladesh and Pakistan but which subsequently drifted or experienced downward allele frequency selection pressure within Asia resulting in a similar frequency in Brazil Amerindian (table 6), but had occurred pre-migration. The reason for this is unknown.

The genes we have implicated have known functions that span biological processes that could potentially influence reproductive or survival fitness in different and fascinating ways. *CIITA* regulates MHC class II gene transcription and has been called “the master control factor” for expression of these genes. *CIITA* has been implicated in immune function through association with autoimmune diseases or very recently with leprosy (Liu et al. 2015). The gene complex including *CIITA* and neighboring *DEXI/CLEC16A* has been shown to be associated with multiple autoimmune diseases (Bronson et al. 2011; Gyllenberg et al. 2012, 2014; Leikfoss et al. 2015). *DOCK2* is predominantly expressed in hematopoietic cells, regulates migration and activation of neutrophils through Rac activation (Nishikimi et al. 2013) and is associated with early-onset invasive infections (Dobbs et al. 2015). *ADAMTS9* has been shown to be associated with body fat distribution (Liu et al. 2013) and other anthropometry/metabolic traits including type 2 diabetes (Zeggini et al. 2008; Heid et al. 2010; Randall et al. 2013), as well as age-related macular degeneration (Fritsche et al. 2013) and other traits. *ARHGAP5* is one of the RhoGTPase family important in embryonic development (Heckman et al. 2007) and in modulating myometrial contractility in uterine smooth muscle, including during pregnancy (O'Brien et al. 2008). *SLC28A1* codes for a concentrative nucleoside transporter primarily recovering pyrimidines from urine in kidney (Elwi et al. 2006), but may also have a role in immunity and macrophage activation (Loffler et al. 2007).

Our unsupervised analysis of genetic ancestry within our North-Eastern Brazil samples showed that an admixture model of three continental populations, Africa, Europe, and Amerindian, was sufficient to explain the most important ancestral structure, although if we had included other diverse Latin American samples in our admixture and PCA, we undoubtedly would have found finer structure of admixture (Johnson et al. 2011; Moreno-Estrada et al. 2013, 2014) but this was not the goal of the study, and would have been problematic for accurate supervised ancestry estimation at fine resolution. The unsupervised admixture analysis also showed that the African component was closest to African Americans in the US South-West (ASW) and African Caribbeans in Barbados (ACB) but this was most likely due to imperfect partitioning of genetic variance between admixing continental components and European admixture retained within the inferred African component in the absence of supervising proxy samples in the ADMIXTURE analysis, rather than significant recent differentiation (Bhatia et al. 2014). Among populations in Africa, the unsupervised African component was actually most similar to the Luhya in Kenya, but this was also probably biased by similarity to older East African variation through residual European or Amerindian variation. The Africa-centric PCA clearly showed that the recent North-Eastern Brazil admixture arose from a population genetically closer to the West African Yoruba/Esan populations near the Bight of Benin, modern day Nigeria.

This is consistent with the history of slave importation into Brazil. Three broad periods of slave importation are recognized, roughly corresponding to the 16th century (Senegambia/Upper Guinea); 17th century (a switch to importation from Central/West Africa, modern day Congo and Angola); and the 18th century (Mina Coast/Lower Guinea) (Sweet 2003). Before 1700 only 13% of total African slaves came from the Bight of Benin, while in the period 1700–1850 approximately 55% of the slaves that landed in Bahia province—the major landing point in North-Eastern Brazil—came from the Bight (Klein and Luna 2010). The European admixture component is most likely derived from Southern Europe with latter day Spanish being the closest match of the available 1000 Genomes populations, which probably reflects earlier Portuguese influence, although Spaniards and Dutch were also present as explorers and colonial powers.

Inclusion of proxy samples for the components ancestries in a supervised ADMIXTURE analysis resulted in significantly different estimates of individual admixture. Similar results were found in one of the very few and limited genetic studies of North-Eastern Brazilians (768 SNPs), with the use of differing pseudo-ancestral populations (Magalhaes da Silva et al. 2015). We found that as few as 30 proxy samples for each ancestry was sufficient, and of the 1000 Genomes populations, the closest proxies for the predominantly European, Amerindian, and African admixed components were respectively Spanish (IBS); Esan/Yorubans (ESN/YRI); Peruvians (PEL) and a few Mexicans (MXL). The North-Eastern Brazilians had a mean ancestry of 57% European, 20% Amerindian, and 23% African although with considerable ranges of individual ancestries within each component (19–95%, 2–49%, and 4–67%, respectively). The mean ancestries of the five study groups located in coastal Fortaleza, the capital of Ceará state, did not differ, but the mean ancestry in the Recodisa case control study group, which was enrolled in six other noncoastal cities across four North-Eastern states including Ceará, had slightly higher and more variable percentages of European and African ancestries, but about 10% lower and less variable Amerindian ancestry.

The advantages of including proxies to identify components of admixture have described previously but using different methods to assess likely accuracy of ancestral reconstruction (Falush et al. 2003; Tang et al. 2005; Alexander et al. 2009; Alexander and Lange 2011). Although we believe these differences are an improvement in the estimates of admixture proportions, a possible alternative explanation is that supervised ancestry estimates are biased due to European admixture in the Amerindian proxies, and to lesser degree, in the African samples. This illustrates the challenges in accurate admixture analysis and identifying suitable nonadmixed reference or proxy samples for supervised ancestral deconvolution.

Previous studies of urban and regional Brazilian populations have shown that the predominant admixture components are European, African, and Amerindian with systematic variation in the proportions between the five major regions in Brazil, although the accuracy may have been limited by small SNP panels and unsupervised or joint admixture estimation (Ruiz-Linares et al. 2014). More recent genome-wide panels with thousands of samples have found similar structure (Giolo et al. 2012; Kehdy et al. 2015; Lima-Costa et al. 2015) but this is the first study using Brazil samples that has attempted to carefully select best matching proxies and derive supervised genome-wide estimates of admixture components based on ancestral similarity. No other studies have attempted to interrogate the latent ancestry in Brazil for putative selection.

Based on the Fst genetic differentiation results and TREEMIX analyses, we found that the Bengali population is the closest proxy of the Asian 1KG populations for the source ancestral Asian population of migrants into the Americas, believed to be from North-Eastern Siberia (Zakharov et al. 2004; Achilli et al. 2013). The TREEMIX phylogenetic plot (supplementary fig. S8, Supplementary Material online) showed that the Amerindian component is most genetically similar to an admixed Central-East Asian ancestral group containing later admixture between a descendent group of the North-Indian subcontinent clade (PJL, GIH, and BEB) and an older South-East Asian/Japan lineage, albeit with evidence of later reverse gene flow between the Indian and East Asian clades. The topology of the plot is consistent with very recent results from reconstruction of ancestral relationships and admixture events within the Central/East Asia region and an emerging model of an early Southern route migration out of Africa through South Central and Eastern Asia (Duggan and Stoneking 2014; Qin and Stoneking 2015). There is evidence from Bronze Age specimens of Central-East Asian admixture in regions in Siberia proposed as a possible source of the migratory proto-Amerindian population (Hollard et al. 2014), which now seems to have occurred as a single migration wave, approximately 23 kya (Raghavan et al. 2015).

In conclusion, we have identified multiple differentiated regions in the Amerindian ancestral component of the North-Eastern Brazil population, drawn from six separate studies containing SNPs located in genes involved in immune function, metabolism, embryonic development, and other diseases and traits. We recognize that our results could be biased by the genetic panel used as the source of the SNP genotyping data, although the panel is informative for Latin American populations; that we have only investigated the ancestry derived from a single country, albeit one with a high degree of admixture; and that we have not proven that the most differentiated SNPs or genes are functionally under selection. Further work is needed to replicate these findings in other studies and to understand the health implications of the results.

## Materials and Methods

### Study Populations

The genetic samples analyzed in this work were drawn from six cohorts/studies conducted on populations in North-Eastern Brazil and centered on Fortaleza, Ceará state. They have been previously described in detail and will only be reviewed briefly here. The Gonçalves Dias cohort was recruited in the Gonçalves Dias favela in Fortaleza between 1989 and 1993 to study the epidemiology, nutritional impact and causes of persistent diarrhea in early childhood (Lima et al. 2000). The Malnutrition and Enteric Disease Network (Mal-ED) Birth Cohort enrolled 242 children within 17 days of birth between 2010 and 2014; an additional 101 infants recruited under the ICIDR (International Center for Infectious Disease Research) program and evaluated by the same procedures as Mal-ED are included in the cohort. The prospective Mal-ED case-control (MCC) study enrolled 401 children 6–18 months of age between 2010 and 2014. Both Mal-ED study groups were enrolled in Fortaleza (Lima et al. 2014). The Recodisa prospective case-control study enrolled 1200 children aged 2–36 months between 2010 and 2014 from hospitals or clinic facilities in six semiarid countryside cities of North-Eastern Brazil to study the etiology of diarrhea. The cities were Crato (Ceará state), Cajazeiras, Souza, and Patos (Paraíba state), Ouricuri (Pernambuco state), and Picos (Piauí state) and had >50,000 inhabitants in states with >50% area localized inside the Brazilian Semiarid region. Target enrollment was 100 cases and controls from each city.

The Parque Universitário Zinc-Vitamin A clinical trial cohort enrolled 324 children between 2000 and 2006, and the Parque Universitário Zinc-Arginine clinical trial cohort enrolled 349 infants between 2006 and 2010, both from the Parque Universitário favela in Fortaleza (Lima et al. 2013). All families gave informed consent for genetic research into diseases and traits linked to malnutrition. The study protocols were approved by the Federal University of Ceará Committee for Ethics in Research and the University of Virginia Institutional Review Board for Health Sciences Research. Although this study contained incidental analysis of ancestry and anthropology, this was performed in so far as it was required to construct correctly-adjusted statistical tests to identify regions of the genome and SNPs that might be linked to disease susceptibility and to enable future tests of association. All participants were de-identified to the analysis and no interpretation of the ancestry of specific identified participants was performed.

### Genome-Wide Genotyping and Quality Control

Saliva samples from all children were collected using Oragene DNA kit G-250 (DNA Genotek, Ontario, Canada). Briefly, the sample collector was mixed gently and incubated at 50 °C for 1 h in a water bath. Unabsorbed liquid was transferred to a conical 15 mL centrifuge tube and the barrel of a 5 mL disposable syringe, containing collected sponges, was also placed inside the tube and centrifuged at 200 × *g* for 10 min at 20 °C. After centrifugation, the syringes were removed and the DNA was manually extracted from 4.0 mL of Oragene DNA/saliva according to published vendor protocols. All samples were genotyped on the Affymetrix Axiom Latin America Array (LAT-1) with 818,154 SNPs and Indels specifically informative for Hispanic and Latin American populations. A total of 2,119 Brazil sample CEL files were processed using Affymetrix power tools (APT 1.16.0), applying the vendor’s best practices quality control (QC) criteria for samples and SNPs. More details are available in supplementary table S1, Supplementary Material online. After Affymetrix quality control 1,659 samples and 755,801 SNPs were available for genetic ancestry analysis. Additional sample quality control for cryptic relatedness up to degree 2 was performed using KING (Manichaikul et al. 2010), and after removing related and sex-misclassified samples, 1,538 samples remained. Further SNP QC, dropping SNPs with a call rate <99% and/or minor allele frequency (MAF) <5%, resulted in a total autosomal chromosome SNP count of 410,172 SNPs. Finally these SNPs were thinned to reduce residual linkage disequilibrium (LD), so that the maximum inter-SNP r2 was 0.3, resulting in 199,654 SNPs. Plink 1.07 was used for genetic data management and to calculate LD (Purcell et al. 2007).

### 1000 Genomes Project Data and Quality Control

The 1000 Genomes Project (1KG) phase3 release data (version date 2013/05/02) was downloaded (ftp://ftp-trace.ncbi.nih.gov/1000genomes/ftp/release/20130502; last accessed January 30, 2015) and contained 2,504 samples from 26 populations. KING was used to identify residual relatedness up to degree 2, inferring eight parent-offspring, four full sibs and three 2nd degree relative pairs with one family of size 3. After dropping one of the related pairs and filtering SNPs > 1% MAF, the total data set was 2,490 samples × 30.7M variants. Intersection of the post-QC Brazil SNP (MAF ≥0.05) with the 1KG data resulted in 400,150 total autosomal SNPs for locus testing. The merged Brazil low LD data set and 1KG data resulted in 195,090 autosomal SNPs. This data set was used for the Brazil + 1KG joint PCA and supervised admixture analyses.

### Principal Component and Admixture Analysis

Admixture analysis was performed using ADMIXTURE v1.23 in supervised and unsupervised modes as described in the main text, and was run with 10 cross-validation folds (–cv = 10) (Alexander et al. 2009; Alexander and Lange 2011). Principal component analysis was performed using the EIGENSOFT package v5.0.1 (Patterson et al. 2006; Price et al. 2006). Unsupervised analysis used the Brazil post-QC LD-thinned SNP set of 199,654. For supervised analyses, the number of proxy reference samples was constrained to be equal for each component ancestry to reduce the bias in estimation of ancestry composition through different likelihood weighting, or the distortion of principal component axes (McVean 2009).

### Statistical Genetic Analysis

Tests of equality of mean ancestry between the six study groups were performed using Hotelling’s test applied to each pair of study groups testing bivariate equality of means of two independent ancestry proportions (%AMR, %AMR) of the three constrained total. The significance level was adjusted for 15 tests, (alpha = 0.05/15). Genetic differentiation between ancestral admixture components and populations was measured using Hudson’s Fst (Nei 1973; Hudson et al. 1992). This was computed using custom R functions according to the algorithm and estimator described in Bhatia et al. (2013). 95% Confidence intervals for Fst estimates were generated using 10,000 bootstrap resamples and the bootstrap percentile method (Efron and Tibshirani 1993). The genetic differentiation of SNP loci for the Amerindian-specific ancestral component of the Brazil samples was assessed under two scenarios. The branch-specific Fst (Shriver et al. 2004) was computed for each SNP for the Brazil Amerindian admixture component (BRN2) relative to a hypothetical single ancestral population for all three admixture components (ancestral) where:

(1) $$Fst\left(2\;\text{vs}.\;\text{ancestral}\right)=\frac{Fst\left(1\;\text{vs}.\;2\right)+Fst\left(2\;\text{vs}.\;3\right)-Fst(1\;\text{vs}.\;3)}{2}$$

This statistic is monotonically related to the population branch statistic which has an identical form but with scaled population divergence time estimated as T=−log(1−Fst) (Cavalli-Sforza 1969) substituted for each pairwise *Fst*. The second scenario identified the most highly genetically differentiated loci comparing the closest proxy Asian 1KG population, Bengalis in Bangladesh (BEB) to the Brazil Amerindian BRN2(Amr) component, using direct pairwise Fst, BRN2(Amr) vs. BEB). R version 3.0.3 was used for all other statistical analyses (R Core Team 2014).

### Genome Regional Fst Plots

Genome regional plots of extreme Fst differentiation were created using LocusZoom (Pruim et al. 2010). Linkage disequilibrium (*r*^2^) between SNPs was estimated in LocusZoom using 1KG admixed American (AMR) samples (MXL, PUR, CLM, and PEL).

### TREEMIX Analysis

The relationship between the Amerindian component BRN(Amr) and 1KG Asian populations was analyzed using TREEMIX 1.12 (Pickrell and Pritchard 2012). We included the Yorubans (YRI) as an outgroup and varied the number of migrations from 0 to 8. The BRN2(Amr) genotype counts were estimated as 2 × mean proportion of BRN2(Amr) admixture (0.2) × ADMIXTURE estimated allele frequency, rounded to the nearest integer. We compared the plots of residuals and tested the change in final composite log maximum likelihood using 10,000 bootstrap replicates of the SNPs with the same seed to estimate the bias-corrected 95% confidence interval on the log likelihood (-bootstrap -seed options in TREEMIX). We compared the 1-sided 95% confidence interval test of the model log likelihood with k migration events vs. *k* − 1 events to identify the most parsimonious migration model at which further increase in the migration parameter led to insignificant improvement in the maximum likelihood.

## Supplementary Material

Supplementary tables S1–S5 and figures S1–S28 are available at *Molecular Biology and Evolution* online.

## Supplementary Material

Supplementary Data
