# Supplementary material for: Genome-Wide Analysis in Brazilians Reveals Highly Differentiated Native American Genome Regions
Source: Mol Biol Evol. 2017 Jan 18;34(3):559–74. doi: 10.1093/molbev/msw249 (PMC5430616; doi:10.1093/molbev/msw249)
Supplement: Supplementary Data [file msw249_Supp.zip › Supplementary Figures S1-S14.pdf]

Supplementary Figure Legends

Supplementary Figure S1. Results of unsupervised admixture analysis of the Brazil samples to select the best-fitting latent population structure (K)

The x-axis shows the number of latent sub-populations (K) tested and the y-axis shows the Admixture cross-validation error for each assumed K value using 10-fold prediction. The K corresponding to the minimum admixture CV value is preferred, here K=3.

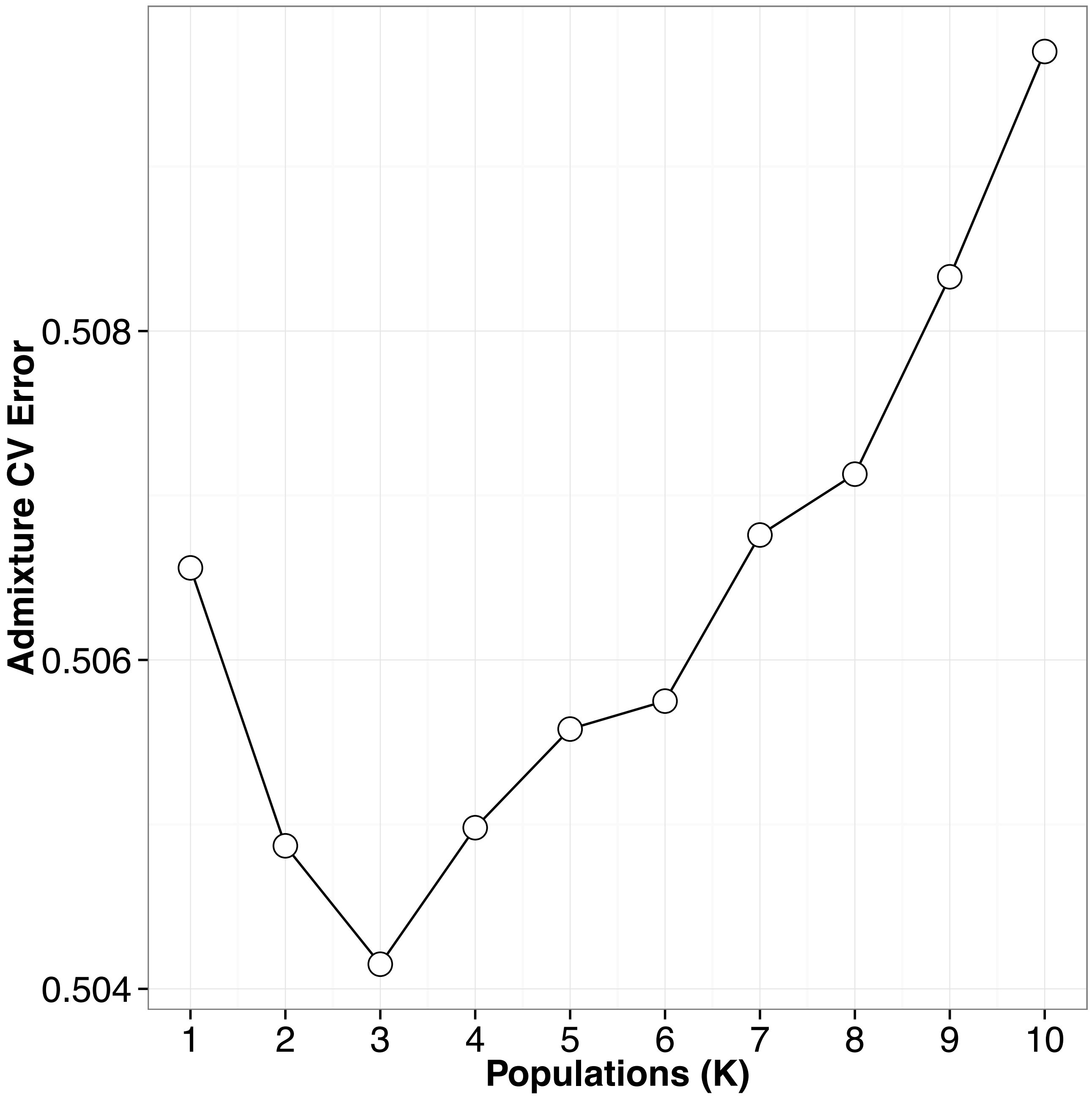

Admixture CV Error
0.504
0.506
0.508
1
2
3
4
5
6
7
8
9
10
Populations (K)

Supplementary Figure S2. Inference of the optimal number of ancestral proxy samples to include in a supervised admixture analysis

Panel a. shows the root mean square standard error of admixture proportions per Brazil sample (BRN) plotted against the number of ancestral samples included in each of the 3 current day ancestral proxy populations for the supervised inference of admixture proportions. The minimum root mean square of the ancestry proportions standard error per sample is at N=30, although the minimum is shallow and broad.

Panel b. shows how the predominant ancestry of the 3 inferred populations change for the Brazil samples changes relative to the number of ancestral proxy samples included. The inferred population ancestries are labeled 1,2,3 here rather than whether they are predominantly similar to European, African, or Amerindian. The initial population assignments for unsupervised analysis (N=0) change in assignment as N=10 samples are included and then remain stable to N=50 before exchanging at N=100. This confirms that the population assignments are stable in the N=10-50 range corresponding to the broad valley in the plot shown in panel a.

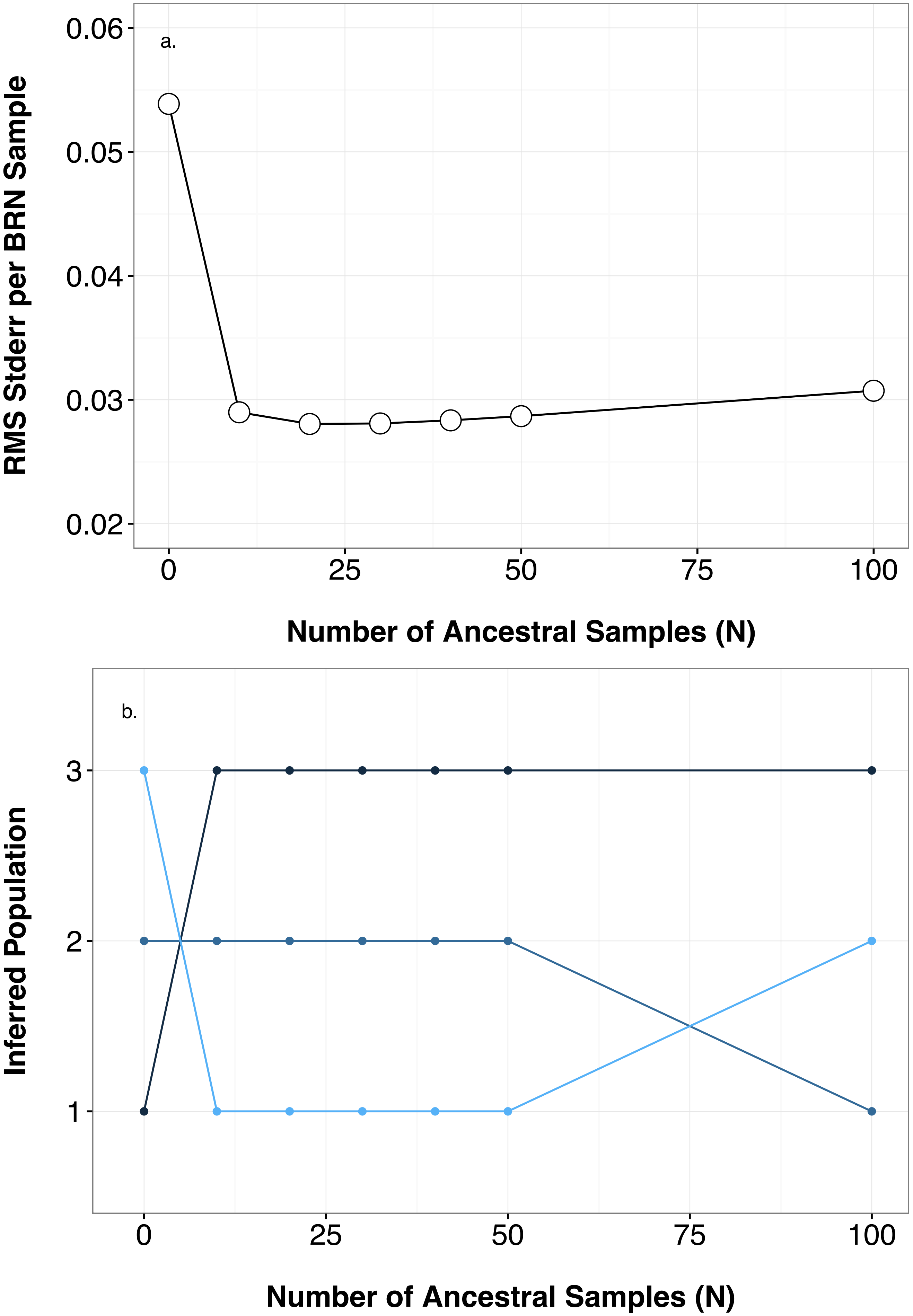

a.
RMS Stderr per BRN Sample
0.06
0.05
0.04
0.03
0.02
0
25
50
75
100
Number of Ancestral Samples (N)
b.
Inferred Population
3
2
1
0
25
50
75
100
Number of Ancestral Samples (N)

Supplementary Figure S3. The most highly differentiated region in the human genome for variation accrued along the Amerindian branch in the Brazil samples from the inferred root of the 3 continental ancestries.

The plot was generated from the UCSC Genome Browser using the hg19 human genome assembly (http://genome.ucsc.edu), reference in the main text. The two custom tracks in green at the top of the plot show any regional SNPs within the 500 top ranked SNPs by Fst statistic for either the test of the Amerindian locus-specific branch length (top green track) or the corresponding top 500 SNPs for the test of locus differentiation in the Brazil Amerindian component versus the closest Bengalis in Bangladesh 1KG samples. The top differentiated SNP was rs6498115 in the proximal promoter of the *CIITA* gene.

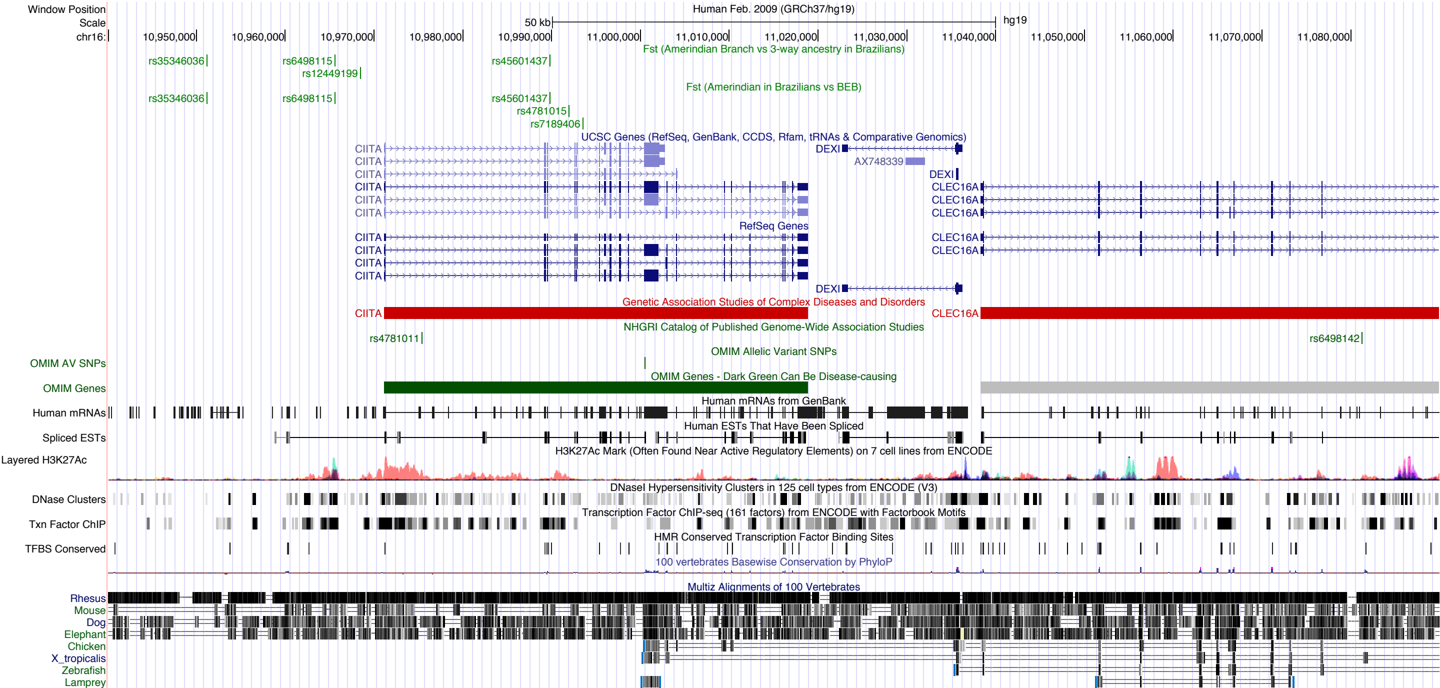

Window Position
Scale
chr16:
Human Feb. 2009 (GRCh37/hg19)
50 kb
hg19
10,950,000
10,960,000
10,970,000
10,980,000
10,990,000
11,000,000
11,010,000
11,020,000
11,030,000
11,040,000
11,050,000
11,060,000
11,070,000
11,080,000
Fst (Amerindian Branch vs 3-way ancestry in Brazilians)
rs35346036
rs6498115
rs12449199
rs45601437
Fst (Amerindian in Brazilians vs BEB)
rs35346036
rs6498115
rs45601437
rs4781015
rs7189406
UCSC Genes (RefSeq, GenBank, CCDS, Rfam, tRNAs & Comparative Genomics)
CIITA
DEXI
AX748339
CLEC16A
RefSeq Genes
Genetic Association Studies of Complex Diseases and Disorders
NHGRI Catalog of Published Genome-Wide Association Studies
rs4781011
rs6498142
OMIM AV SNPs
OMIM Allelic Variant SNPs
OMIM Genes
OMIM Genes - Dark Green Can Be Disease-causing
Human mRNAs
Human mRNAs from GenBank
Spliced ESTs
Human ESTs That Have Been Spliced
Layered H3K27Ac
H3K27Ac Mark (Often Found Near Active Regulatory Elements) on 7 cell lines from ENCODE
DNase Clusters
DNaseI Hypersensitivity Clusters in 125 cell types from ENCODE (V3)
Txn Factor ChIP
Transcription Factor ChIP-seq (161 factors) from ENCODE with Factorbook Motifs
TFBS Conserved
HMR Conserved Transcription Factor Binding Sites
100 vertebrates Basewise Conservation by PhyloP
Multiz Alignments of 100 Vertebrates
Rhesus
Mouse
Dog
Elephant
Chicken
X_tropicalis
Zebrafish
Lamprey

Supplementary Figure S4. ***SMC6*** **differentiated genome region**

The second most highly differentiated region in the human genome for variation accrued along the Amerindian branch in the Brazil samples from the inferred root of the 3 continental ancestries. The plot was generated similarly to Supplementary Figure S3 and as described in the text. The most highly differentiated SNP in this region was rs1834619 in an intron of the structural maintenance of chromosomes 6 (*SMC6)* gene.

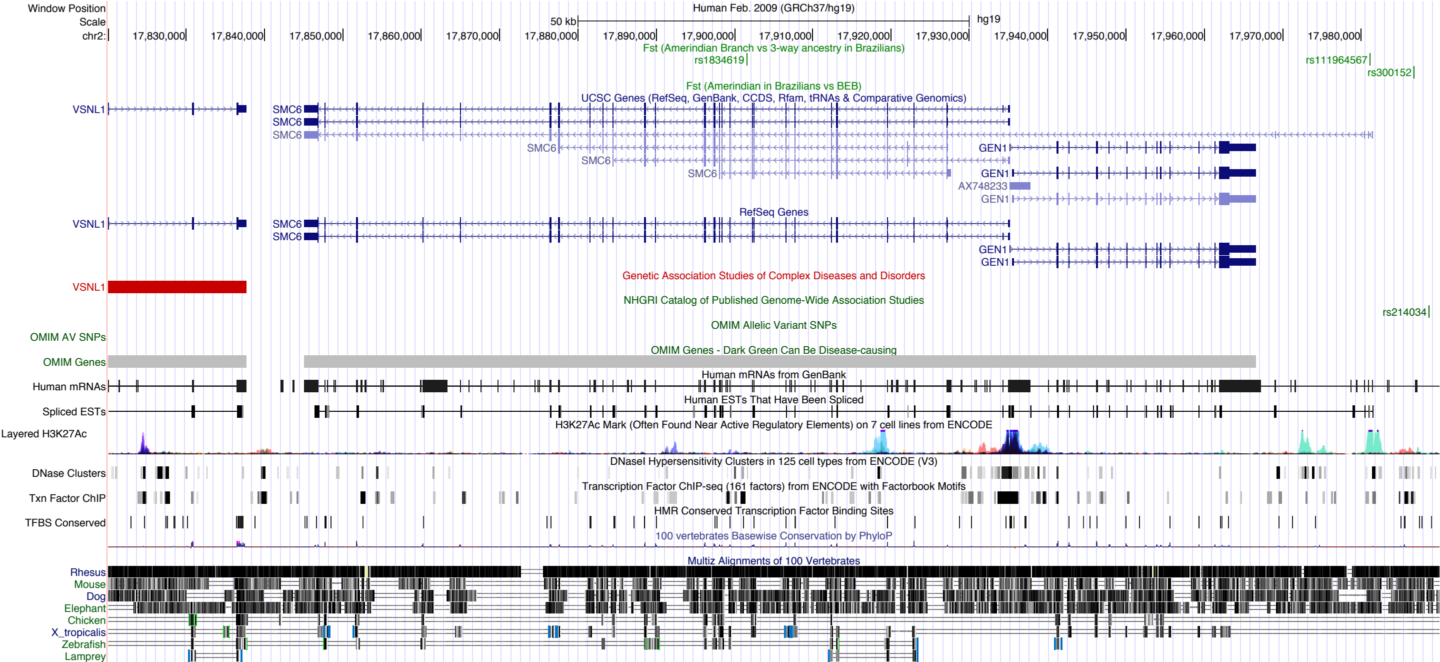
Window Position
Scale
chr2:
Human Feb. 2009 (GRCh37/hg19)
50 kb
hg19
17,830,000
17,840,000
17,850,000
17,860,000
17,870,000
17,880,000
17,890,000
17,900,000
17,910,000
17,920,000
17,930,000
17,940,000
17,950,000
17,960,000
17,970,000
17,980,000
Fst (Amerindian Branch vs 3-way ancestry in Brazilians)
rs1834619
rs111964567
rs300152
Fst (Amerindian in Brazilians vs BEB)
UCSC Genes (RefSeq, GenBank, CCDS, Rfam, tRNAs & Comparative Genomics)
VSNL1
SMC6
SMC6
SMC6
SMC6
SMC6
SMC6
GEN1
GEN1
AX748233
GEN1
RefSeq Genes
VSNL1
SMC6
SMC6
GEN1
GEN1
Genetic Association Studies of Complex Diseases and Disorders
VSNL1
NHGRI Catalog of Published Genome-Wide Association Studies
rs214034
OMIM Allelic Variant SNPs
OMIM AV SNPs
OMIM Genes - Dark Green Can Be Disease-causing
OMIM Genes
Human mRNAs from GenBank
Human mRNAs
Human ESTs That Have Been Spliced
Spliced ESTs
H3K27Ac Mark (Often Found Near Active Regulatory Elements) on 7 cell lines from ENCODE
Layered H3K27Ac
DNaseI Hypersensitivity Clusters in 125 cell types from ENCODE (V3)
DNase Clusters
Transcription Factor ChIP-seq (161 factors) from ENCODE with Factorbook Motifs
Txn Factor ChIP
HMR Conserved Transcription Factor Binding Sites
TFBS Conserved
100 vertebrates Basewise Conservation by PhyloP
Multiz Alignments of 100 Vertebrates
Rhesus
Mouse
Dog
Elephant
Chicken
X_tropicalis
Zebrafish
Lamprey

Supplementary Figure S5. ***KLHL29*** **differentiated genome region**

The third most highly differentiated region in the human genome for variation accrued along the Amerindian branch in the Brazil samples from the inferred root of the 3 continental ancestries. The plot was generated similarly to Supplementary Figure S3 and as described in the text. The most highly differentiated SNP in this region was rs2288697 in an intron of the kelch like member 29 (*KLHL29)* gene.

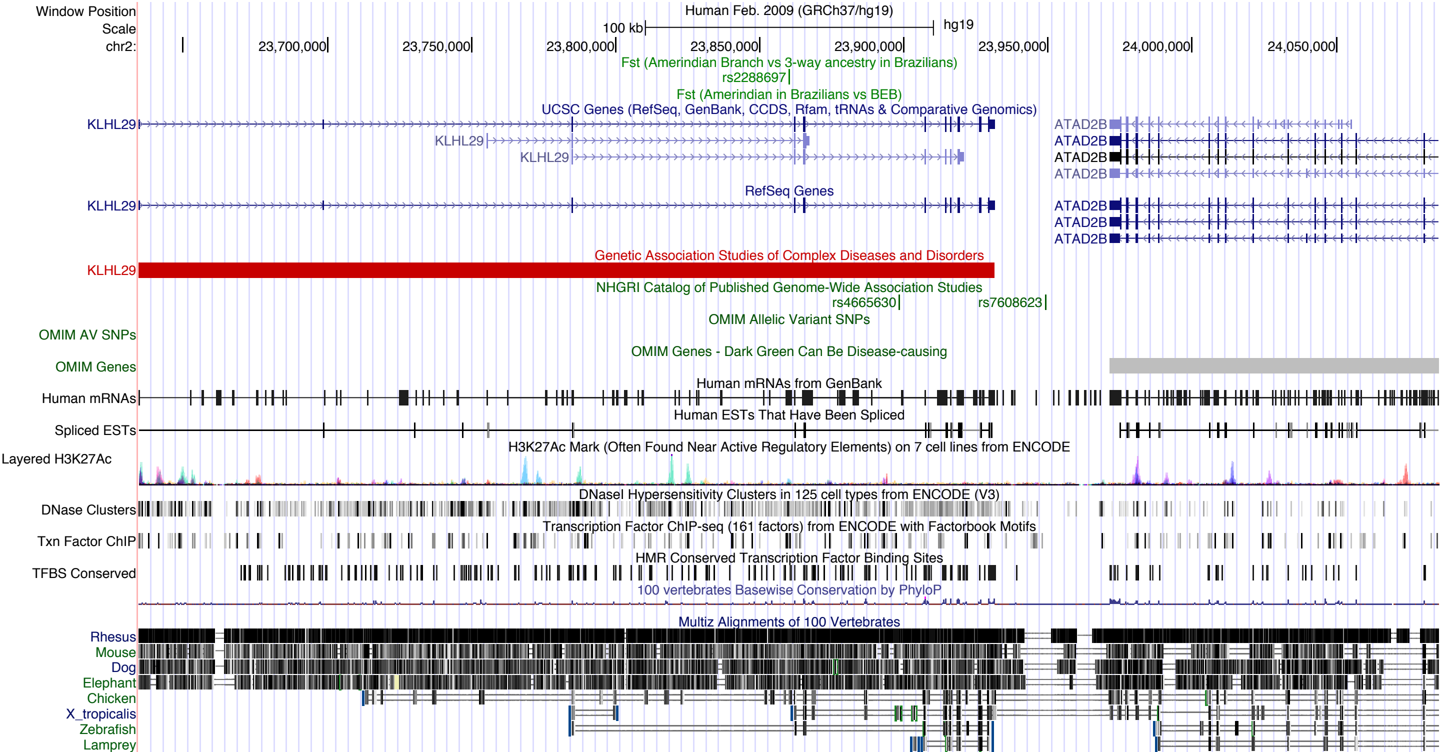
Window Position
Human Feb. 2009 (GRCh37/hg19)
Scale
100 kb
hg19
chr2:
23,700,000
23,750,000
23,800,000
23,850,000
23,900,000
23,950,000
24,000,000
24,050,000
Fst (Amerindian Branch vs 3-way ancestry in Brazilians)
rs2288697
Fst (Amerindian in Brazilians vs BEB)
UCSC Genes (RefSeq, GenBank, CCDS, Rfam, tRNAs & Comparative Genomics)
KLHL29
ATAD2B
RefSeq Genes
Genetic Association Studies of Complex Diseases and Disorders
NHGRI Catalog of Published Genome-Wide Association Studies
rs4665630
rs7608623
OMIM Allelic Variant SNPs
OMIM AV SNPs
OMIM Genes - Dark Green Can Be Disease-causing
OMIM Genes
Human mRNAs from GenBank
Human mRNAs
Human ESTs That Have Been Spliced
Spliced ESTs
H3K27Ac Mark (Often Found Near Active Regulatory Elements) on 7 cell lines from ENCODE
Layered H3K27Ac
DNaseI Hypersensitivity Clusters in 125 cell types from ENCODE (V3)
DNase Clusters
Transcription Factor ChIP-seq (161 factors) from ENCODE with Factorbook Motifs
Txn Factor ChIP
HMR Conserved Transcription Factor Binding Sites
TFBS Conserved
100 vertebrates Basewise Conservation by PhyloP
Multiz Alignments of 100 Vertebrates
Rhesus
Mouse
Dog
Elephant
Chicken
X_tropicalis
Zebrafish
Lamprey

Supplementary Figure S6. ***Chromosome 16, 75.8MBase*** **differentiated genome region**

The fourth most highly differentiated region in the human genome for variation accrued along the Amerindian branch in the Brazil samples from the inferred root of the 3 continental ancestries. The plot was generated similarly to Supplementary Figure S3 and as described in the text. The most highly differentiated SNP in this region was rs2866065 in an inter-genic region of chromosome 16.

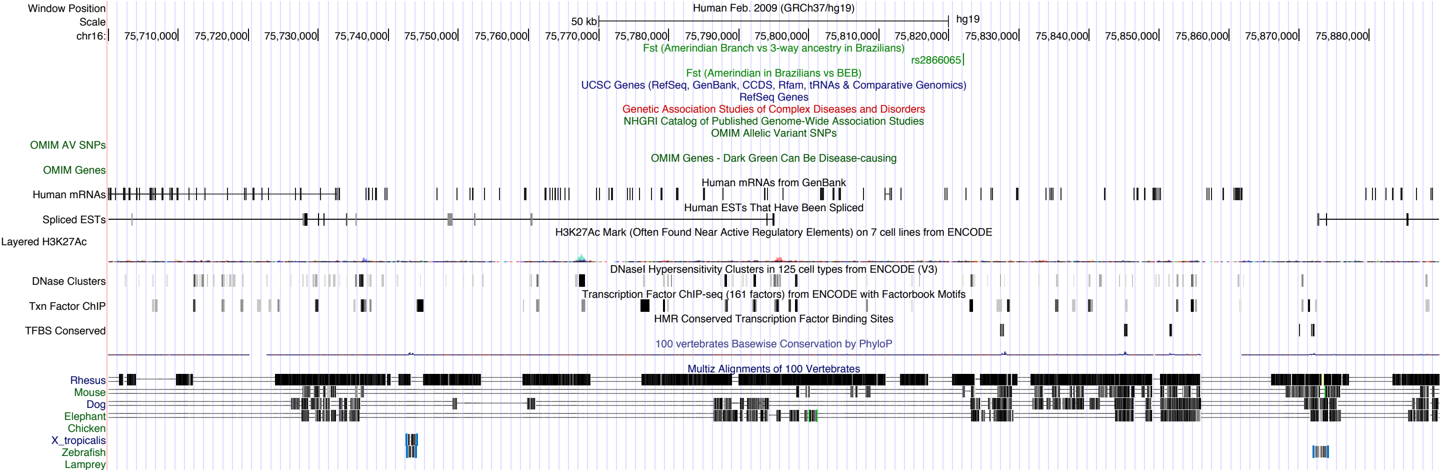
Window Position
Human Feb. 2009 (GRCh37/hg19)
Scale
50 kb
hg19
chr16:
75,710,000
75,720,000
75,730,000
75,740,000
75,750,000
75,760,000
75,770,000
75,780,000
75,790,000
75,800,000
75,810,000
75,820,000
75,830,000
75,840,000
75,850,000
75,860,000
75,870,000
75,880,000
Fst (Amerindian Branch vs 3-way ancestry in Brazilians)
rs2866065
Fst (Amerindian in Brazilians vs BEB)
UCSC Genes (RefSeq, GenBank, CCDS, Rfam, tRNAs & Comparative Genomics)
RefSeq Genes
Genetic Association Studies of Complex Diseases and Disorders
NHGRI Catalog of Published Genome-Wide Association Studies
OMIM Allelic Variant SNPs
OMIM AV SNPs
OMIM Genes
OMIM Genes - Dark Green Can Be Disease-causing
Human mRNAs from GenBank
Human mRNAs
Human ESTs That Have Been Spliced
Spliced ESTs
H3K27Ac Mark (Often Found Near Active Regulatory Elements) on 7 cell lines from ENCODE
Layered H3K27Ac
DNaseI Hypersensitivity Clusters in 125 cell types from ENCODE (V3)
DNase Clusters
Transcription Factor ChIP-seq (161 factors) from ENCODE with Factorbook Motifs
Txn Factor ChIP
HMR Conserved Transcription Factor Binding Sites
TFBS Conserved
100 vertebrates Basewise Conservation by PhyloP
Multiz Alignments of 100 Vertebrates
Rhesus
Mouse
Dog
Elephant
Chicken
X_tropicalis
Zebrafish
Lamprey

Supplementary Figure S7. *MEIS2* differentiated genome region

The fifth most highly differentiated region in the human genome for variation accrued along the Amerindian branch in the Brazil samples from the inferred root of the 3 continental ancestries. The plot was generated similarly to Supplementary Figure S3 and as described in the text. The most highly differentiated SNP in this region was rs16964480 in an intron of the Meis homeobox 2 (*MEIS2*) gene.

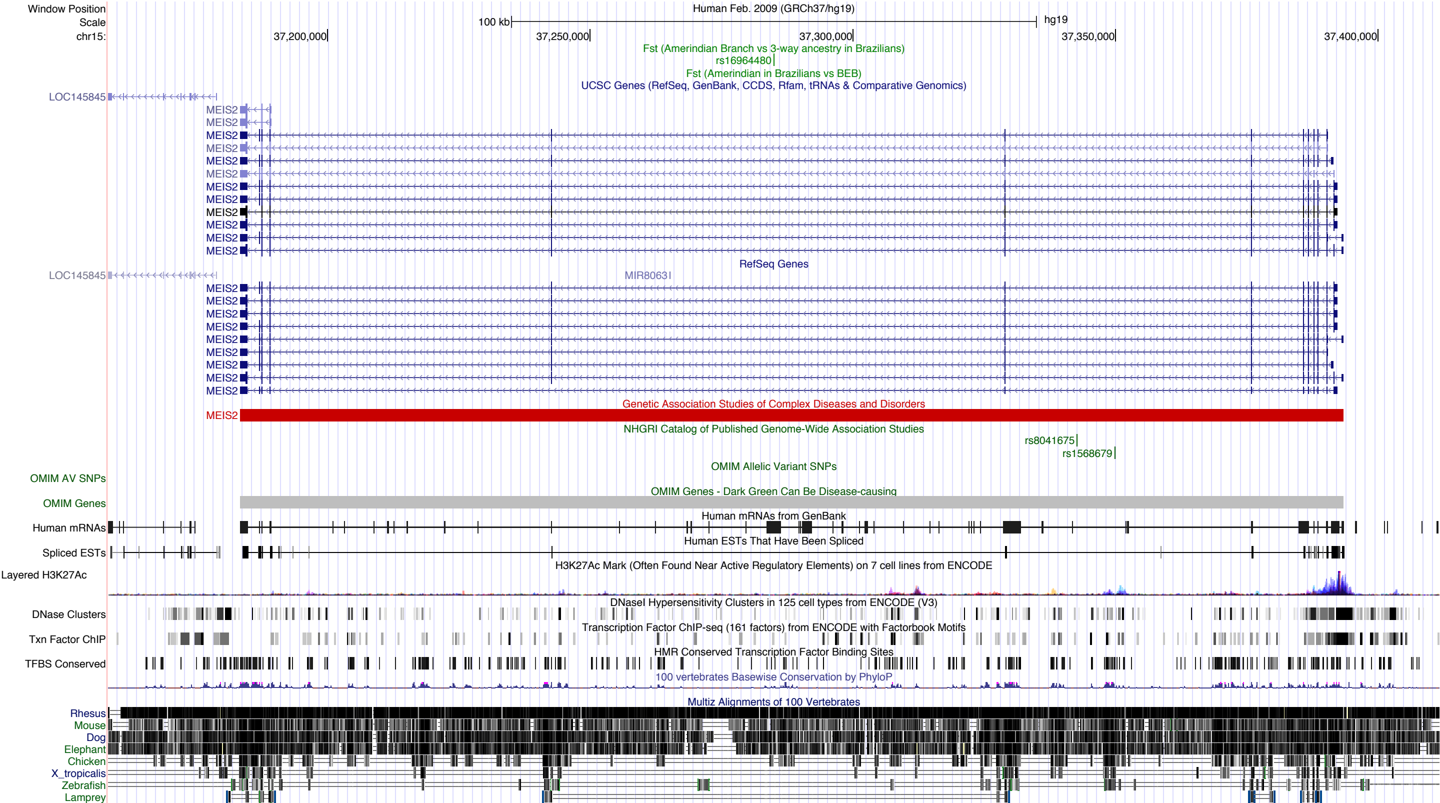
Window Position
Scale
chr15:
Human Feb. 2009 (GRCh37/hg19)
100 kb
hg19
37,200,000
37,250,000
37,300,000
37,350,000
37,400,000
Fst (Amerindian Branch vs 3-way ancestry in Brazilians)
rs16964480
Fst (Amerindian in Brazilians vs BEB)
UCSC Genes (RefSeq, GenBank, CCDS, Rfam, tRNAs & Comparative Genomics)
LOC145845
MEIS2
RefSeq Genes
MIR8063
Genetic Association Studies of Complex Diseases and Disorders
NHGRI Catalog of Published Genome-Wide Association Studies
rs8041675
rs1568679
OMIM Allelic Variant SNPs
OMIM AV SNPs
OMIM Genes - Dark Green Can Be Disease-causing
OMIM Genes
Human mRNAs from GenBank
Human mRNAs
Human ESTs That Have Been Spliced
Spliced ESTs
H3K27Ac Mark (Often Found Near Active Regulatory Elements) on 7 cell lines from ENCODE
Layered H3K27Ac
DNaseI Hypersensitivity Clusters in 125 cell types from ENCODE (V3)
DNase Clusters
Transcription Factor ChIP-seq (161 factors) from ENCODE with Factorbook Motifs
Txn Factor ChIP
HMR Conserved Transcription Factor Binding Sites
TFBS Conserved
100 vertebrates Basewise Conservation by PhyloP
Multiz Alignments of 100 Vertebrates
Rhesus
Mouse
Dog
Elephant
Chicken
X_tropicalis
Zebrafish
Lamprey

Supplementary Figure S8. Plot of log likelihood differences between consecutive TREEMIX models containing N versus N-1 migration events.

The y axis shows the difference in the log likelihood for corresponding TREEMIX models from the same bootstrap set plotted against the contrasting models of N versus N-1 migration events. The 1-sided 95% bias-corrected bootstrap confidence interval is shown for each contrast (5th percentile to 100th percentile) while the numbers below each point/interval show the value of the lower 5th percentile log likelihood difference. The inset shows the migration models from 3 vs 2 to 8 vs 7 on expanded vertical scale.

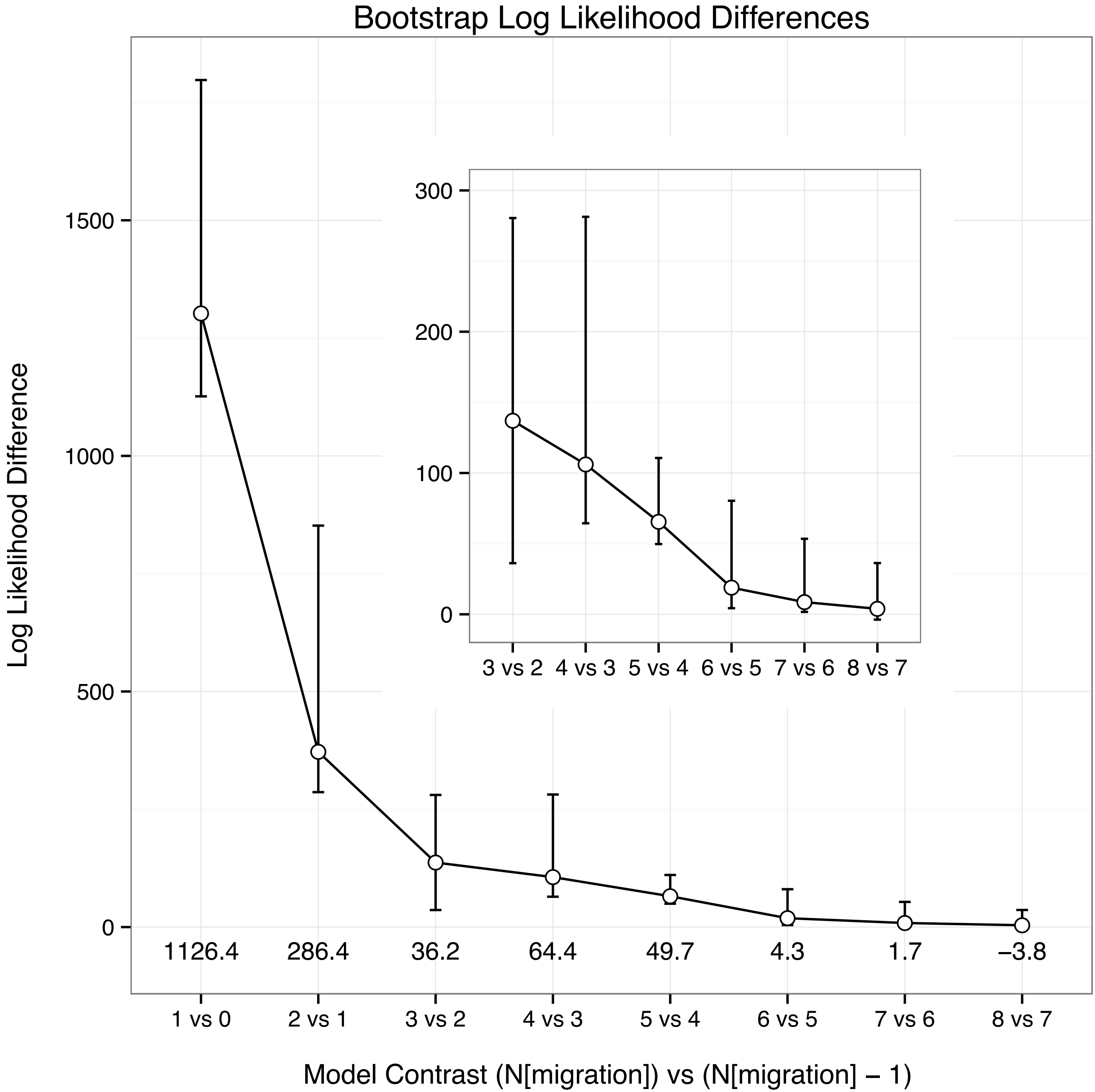

Bootstrap Log Likelihood Differences
Log Likelihood Difference
1500
1000
500
0
300
200
100
0
3 vs 2
4 vs 3
5 vs 4
6 vs 5
7 vs 6
8 vs 7
1126.4
286.4
36.2
64.4
49.7
4.3
1.7
–3.8
1 vs 0
2 vs 1
3 vs 2
4 vs 3
5 vs 4
6 vs 5
7 vs 6
8 vs 7
Model Contrast (N[migration]) vs (N[migration] – 1)

Supplementary Figure S9. Heatmap plot of the residuals from the TREEMIX population ancestral tree analysis incorporating 5 migration events.

The color of each heatmap tile represents the residual covariance for that pair of populations in standard error units.

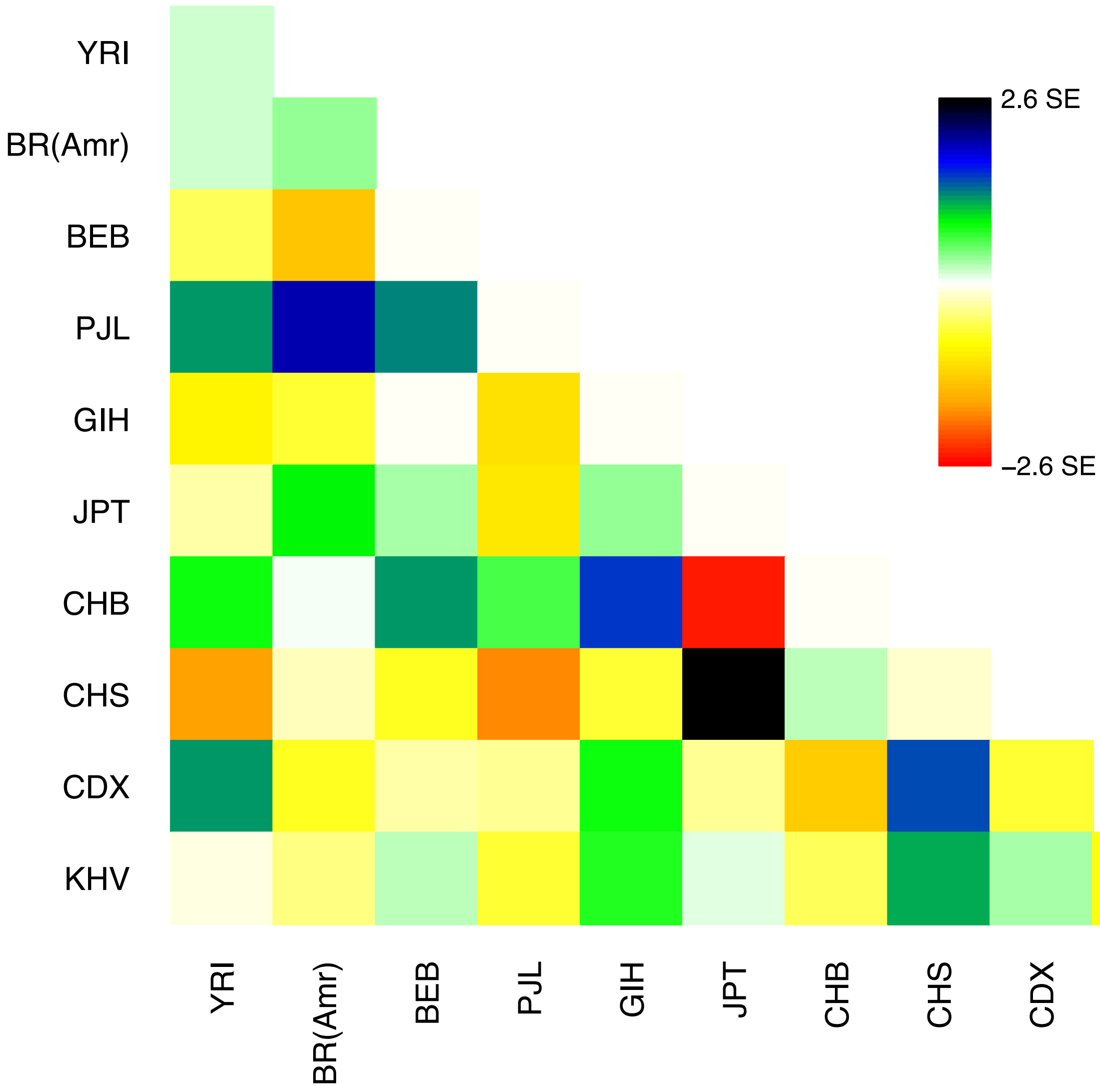
YRI
BR(Amr)
BEB
PJL
GIH
JPT
CHB
CHS
CDX
KHV
2.6 SE
−2.6 SE

Supplementary Figure S10. TREEMIX population ancestral tree model for the Asian 1KG populations and Amerindian component incorporating 5 migration events.

The figure shows the best fitting tree conditioned on 5 migration events, based on the TREEMIX heuristic search and maximum composite likelihood, with inferred ancestral population splits (black bifurcating lines) and migration event arrows based on migration weight. YRI is the Yoruban 1KG outgroup, and BR(Amr) is the reconstructed Brazilian Amerindian population.

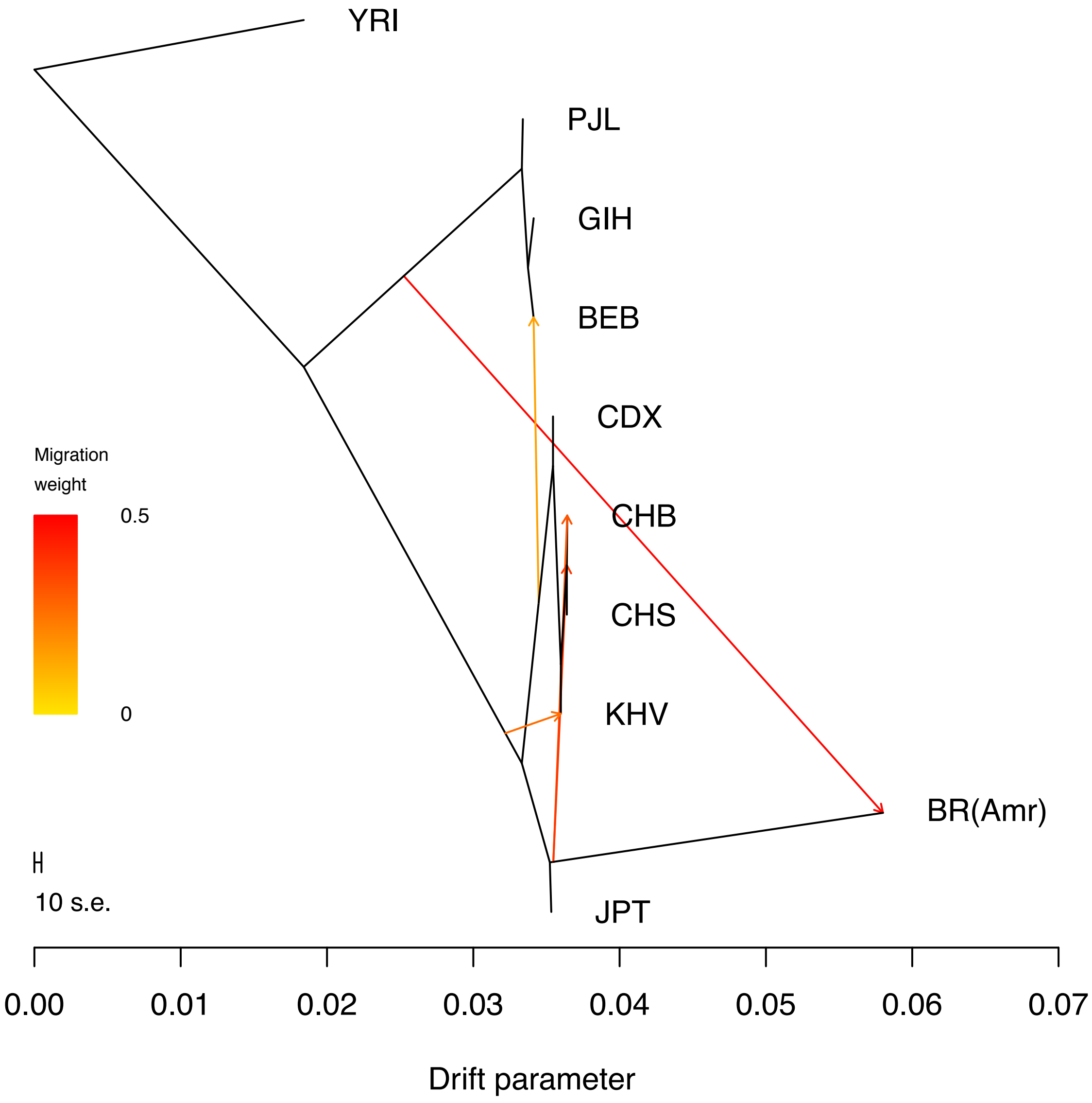

YRI
PJL
GIH
BEB
CDX
CHB
CHS
KHV
BR(Amr)
JPT
Migration weight
0.5
0
10 s.e.
0.00
0.01
0.02
0.03
0.04
0.05
0.06
0.07
Drift parameter

Supplementary Figure S11. The most highly differentiated region in the human genome (*ADAMTS9*) from Fst tests of SNPs in the Brazil Amerindian component of ancestry, versus the most genetically similar 1KG population, the Bengalis in Bangladesh.

The top differentiated SNP was rs7631391, within an intron in the ADAM metallopeptidase with thrombospondin type 1 motif 9 (*ADAMTS9)* gene. The two custom tracks in green at the top of the plot contain plot the regional SNPs that occur within the 500 top ranked SNPs under the two tests of differentiation in this study, and are as described in Supplementary Figure S3.

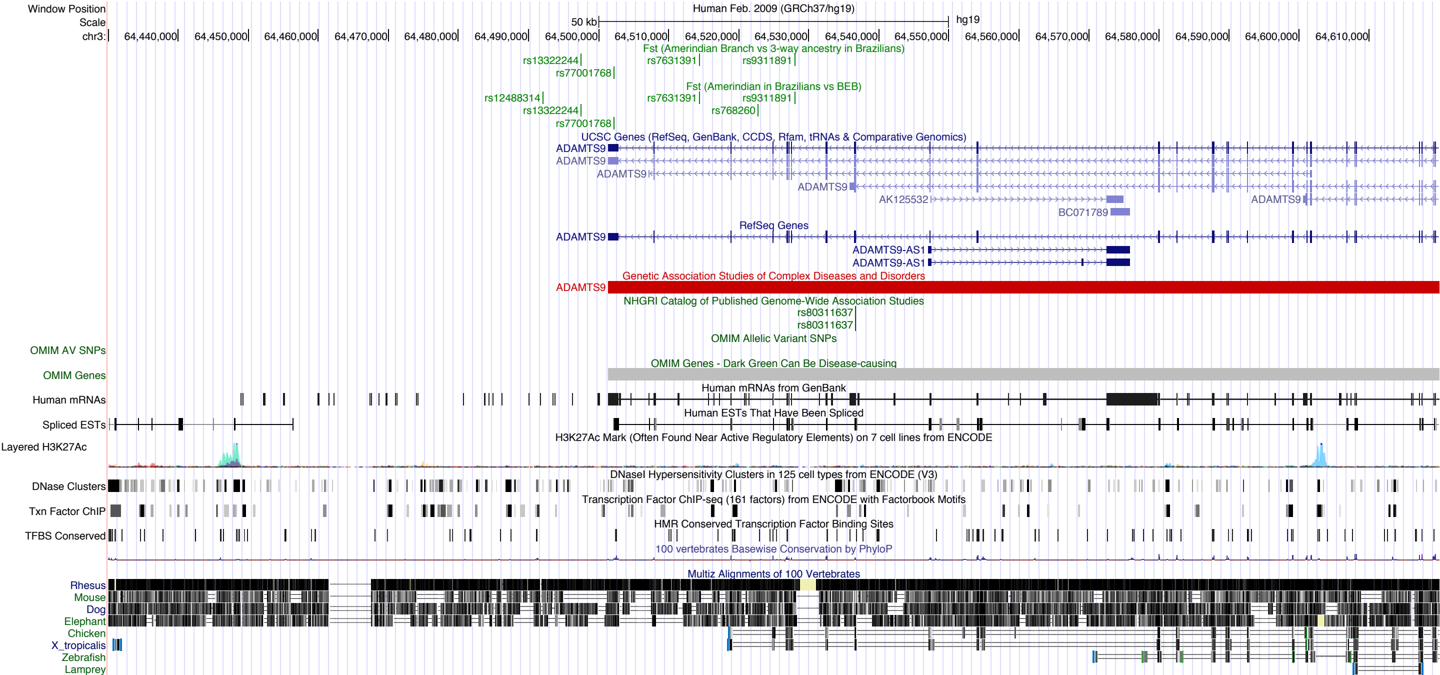

Window Position
Scale
chr3:
Human Feb. 2009 (GRCh37/hg19)
50 kb
hg19
64,440,000
64,450,000
64,460,000
64,470,000
64,480,000
64,490,000
64,500,000
64,510,000
64,520,000
64,530,000
64,540,000
64,550,000
64,560,000
64,570,000
64,580,000
64,590,000
64,600,000
64,610,000
Fst (Amerindian Branch vs 3-way ancestry in Brazilians)
rs13322244
rs7631391
rs9311891
rs77001768
Fst (Amerindian in Brazilians vs BEB)
rs12488314
rs7631391
rs9311891
rs13322244
rs768260
rs77001768
UCSC Genes (RefSeq, GenBank, CCDS, Rfam, tRNAs & Comparative Genomics)
ADAMTS9
ADAMTS9
ADAMTS9
ADAMTS9
AK125532
ADAMTS9
BC071789
RefSeq Genes
ADAMTS9
ADAMTS9-AS1
ADAMTS9-AS1
Genetic Association Studies of Complex Diseases and Disorders
ADAMTS9
NHGRI Catalog of Published Genome-Wide Association Studies
rs80311637
rs80311637
OMIM Allelic Variant SNPs
OMIM AV SNPs
OMIM Genes - Dark Green Can Be Disease-causing
OMIM Genes
Human mRNAs from GenBank
Human mRNAs
Human ESTs That Have Been Spliced
Spliced ESTs
H3K27Ac Mark (Often Found Near Active Regulatory Elements) on 7 cell lines from ENCODE
Layered H3K27Ac
DNaseI Hypersensitivity Clusters in 125 cell types from ENCODE (V3)
DNase Clusters
Transcription Factor ChIP-seq (161 factors) from ENCODE with Factorbook Motifs
Txn Factor ChIP
HMR Conserved Transcription Factor Binding Sites
TFBS Conserved
100 vertebrates Basewise Conservation by PhyloP
Multiz Alignments of 100 Vertebrates
Rhesus
Mouse
Dog
Elephant
Chicken
X_tropicalis
Zebrafish
Lamprey

Supplementary Figure S12. *DOCK2* differentiated genome region

The second most highly differentiated region in the human genome for variation accrued in the Brazil Amerindian component compared to the 1KG BEB population. The plot was generated similarly to Figure 5 as described in the text. The most highly differentiated SNP in this region was rs77594147 in an intron of dedicator of cytokinesis 2 (*DOCK2)* gene.

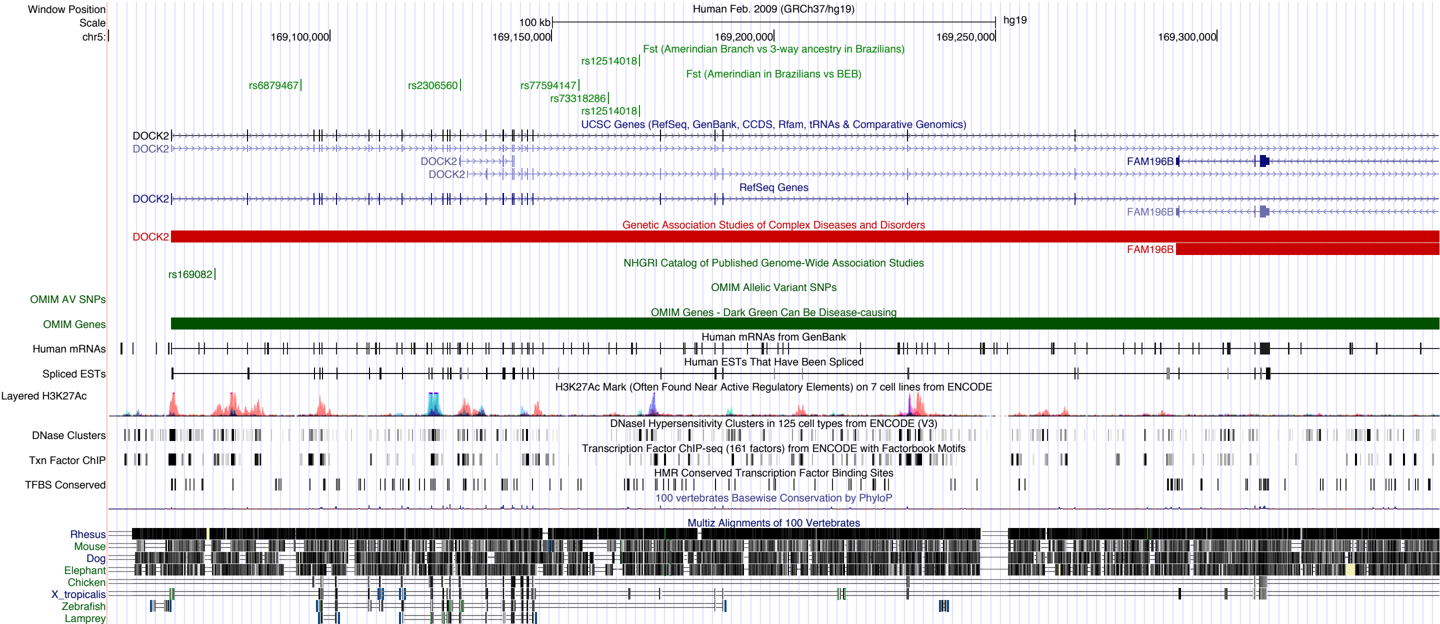

Window Position
Scale
chr5:
Human Feb. 2009 (GRCh37/hg19)
100 kb
hg19
169,100,000
169,150,000
169,200,000
169,250,000
169,300,000
Fst (Amerindian Branch vs 3-way ancestry in Brazilians)
rs12514018
Fst (Amerindian in Brazilians vs BEB)
rs6879467
rs2306560
rs77594147
rs73318286
rs12514018
UCSC Genes (RefSeq, GenBank, CCDS, Rfam, tRNAs & Comparative Genomics)
DOCK2
DOCK2
DOCK2
DOCK2
FAM196B
RefSeq Genes
DOCK2
FAM196B
Genetic Association Studies of Complex Diseases and Disorders
DOCK2
FAM196B
NHGRI Catalog of Published Genome-Wide Association Studies
rs169082
OMIM Allelic Variant SNPs
OMIM AV SNPs
OMIM Genes - Dark Green Can Be Disease-causing
OMIM Genes
Human mRNAs from GenBank
Human mRNAs
Human ESTs That Have Been Spliced
Spliced ESTs
H3K27Ac Mark (Often Found Near Active Regulatory Elements) on 7 cell lines from ENCODE
Layered H3K27Ac
DNaseI Hypersensitivity Clusters in 125 cell types from ENCODE (V3)
DNase Clusters
Transcription Factor ChIP-seq (161 factors) from ENCODE with Factorbook Motifs
Txn Factor ChIP
HMR Conserved Transcription Factor Binding Sites
TFBS Conserved
100 vertebrates Basewise Conservation by PhyloP
Multiz Alignments of 100 Vertebrates
Rhesus
Mouse
Dog
Elephant
Chicken
X_tropicalis
Zebrafish
Lamprey

Supplementary Figure S13. ***SLC28A1*** **differentiated genome region**

The fourth distinct most highly differentiated region in the human genome for variation accrued in the Brazil Amerindian component compared to the 1KG BEB population. The plot was generated similarly to Figure 5 as described in the text. The most highly differentiated SNP in this region was rs28649017 in an intron of solute carrier family 28 (concentrative nucleoside transporter), member 1 (*SLC28A1)* gene.

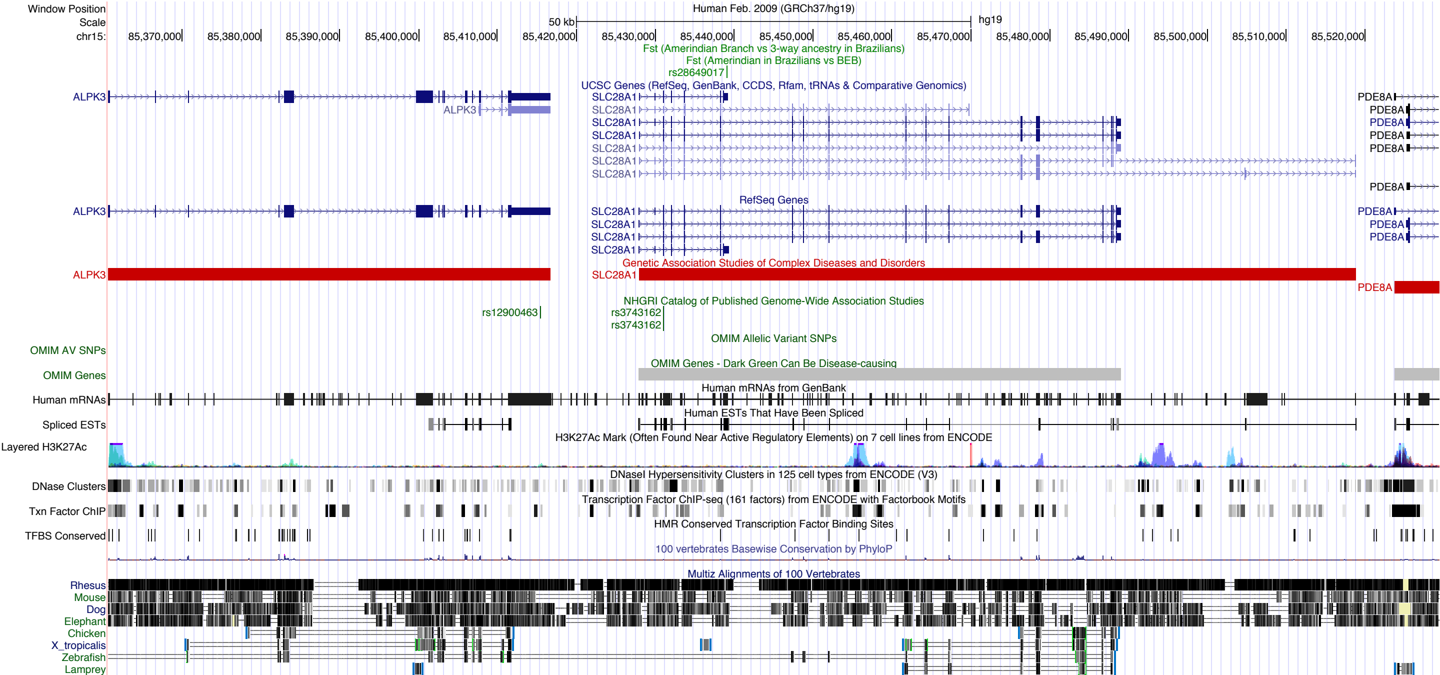

Window Position
Scale
chr15:
Human Feb. 2009 (GRCh37/hg19)
50 kb
hg19
85,370,000
85,380,000
85,390,000
85,400,000
85,410,000
85,420,000
85,430,000
85,440,000
85,450,000
85,460,000
85,470,000
85,480,000
85,490,000
85,500,000
85,510,000
85,520,000
Fst (Amerindian Branch vs 3-way ancestry in Brazilians)
Fst (Amerindian in Brazilians vs BEB)
rs28649017
UCSC Genes (RefSeq, GenBank, CCDS, Rfam, tRNAs & Comparative Genomics)
ALPK3
SLC28A1
PDE8A
RefSeq Genes
Genetic Association Studies of Complex Diseases and Disorders
NHGRI Catalog of Published Genome-Wide Association Studies
rs12900463
rs3743162
rs3743162
OMIM Allelic Variant SNPs
OMIM AV SNPs
OMIM Genes - Dark Green Can Be Disease-causing
OMIM Genes
Human mRNAs from GenBank
Human mRNAs
Human ESTs That Have Been Spliced
Spliced ESTs
H3K27Ac Mark (Often Found Near Active Regulatory Elements) on 7 cell lines from ENCODE
Layered H3K27Ac
DNaseI Hypersensitivity Clusters in 125 cell types from ENCODE (V3)
DNase Clusters
Transcription Factor ChIP-seq (161 factors) from ENCODE with Factorbook Motifs
Txn Factor ChIP
HMR Conserved Transcription Factor Binding Sites
TFBS Conserved
100 vertebrates Basewise Conservation by PhyloP
Multiz Alignments of 100 Vertebrates
Rhesus
Mouse
Dog
Elephant
Chicken
X_tropicalis
Zebrafish
Lamprey

Supplementary Figure S14. *ARHGAP5* differentiated genome region

The third most highly differentiated region in the human genome for variation accrued in the Brazil Amerindian component compared to the 1KG BEB population. The plot was generated similarly to Figure 5 as described in the text. The most highly differentiated SNP in this region was rs7151991 approximately 7 Kbases 3' to Rho GTPase activating protein 5 (*ARHGAP5*) gene.

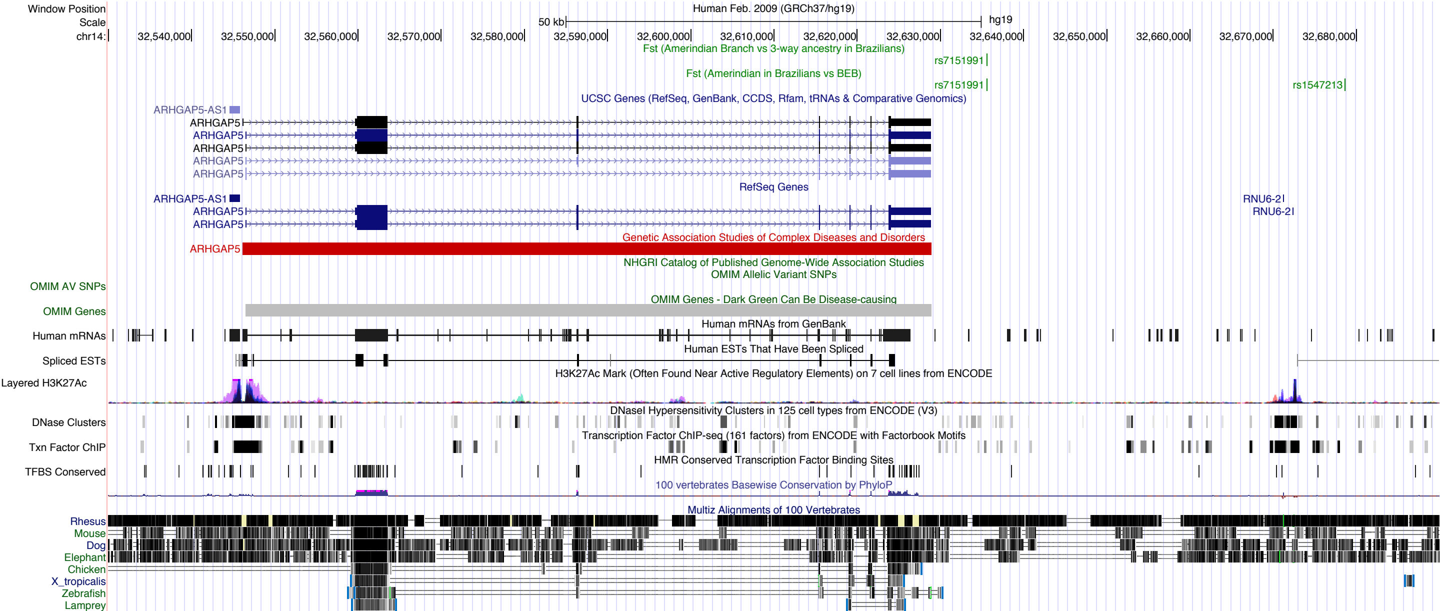

Window Position
Human Feb. 2009 (GRCh37/hg19)
Scale
50 kb
hg19
chr14:
32,540,000
32,550,000
32,560,000
32,570,000
32,580,000
32,590,000
32,600,000
32,610,000
32,620,000
32,630,000
32,640,000
32,650,000
32,660,000
32,670,000
32,680,000
Fst (Amerindian Branch vs 3-way ancestry in Brazilians)
rs7151991
Fst (Amerindian in Brazilians vs BEB)
rs7151991
rs1547213
UCSC Genes (RefSeq, GenBank, CCDS, Rfam, tRNAs & Comparative Genomics)
ARHGAP5-AS1
ARHGAP5
ARHGAP5
ARHGAP5
ARHGAP5
ARHGAP5
RefSeq Genes
ARHGAP5-AS1
ARHGAP5
ARHGAP5
RNU6-21
RNU6-21
Genetic Association Studies of Complex Diseases and Disorders
ARHGAP5
NHGRI Catalog of Published Genome-Wide Association Studies
OMIM Allelic Variant SNPs
OMIM AV SNPs
OMIM Genes - Dark Green Can Be Disease-causing
OMIM Genes
Human mRNAs from GenBank
Human mRNAs
Human ESTs That Have Been Spliced
Spliced ESTs
H3K27Ac Mark (Often Found Near Active Regulatory Elements) on 7 cell lines from ENCODE
Layered H3K27Ac
DNaseI Hypersensitivity Clusters in 125 cell types from ENCODE (V3)
DNase Clusters
Transcription Factor ChIP-seq (161 factors) from ENCODE with Factorbook Motifs
Txn Factor ChIP
HMR Conserved Transcription Factor Binding Sites
TFBS Conserved
100 vertebrates Basewise Conservation by PhyloP
Multiz Alignments of 100 Vertebrates
Rhesus
Mouse
Dog
Elephant
Chicken
X_tropicalis
Zebrafish
Lamprey
