# Supplementary material for: Genome-Wide Analysis in Brazilians Reveals Highly Differentiated Native American Genome Regions
Source: Mol Biol Evol. 2017 Jan 18;34(3):559–74. doi: 10.1093/molbev/msw249 (PMC5430616; doi:10.1093/molbev/msw249)
Supplement: Supplementary Data [file msw249_Supp.zip › Supplementary Figures S15-S28.pdf]

Supplementary Figure Legends S15-S28

Supplementary Figures S15-S28. Principal Component (PC) plots of the first 15 PCs derived from a joint Principal Component Analysis of all Brazil BRN samples (N=1,538) with all 1KG samples (N=2,490)

Principal component analysis was performed using EIGENSOFT. The 14 included figures are plots of all samples for coordinates PC K versus PC K-1 where K =2-15. Plotting legend and color of populations is identical to figures in the main text.

S15 PC2 versus PC1

S16 PC3 versus PC2

etc…

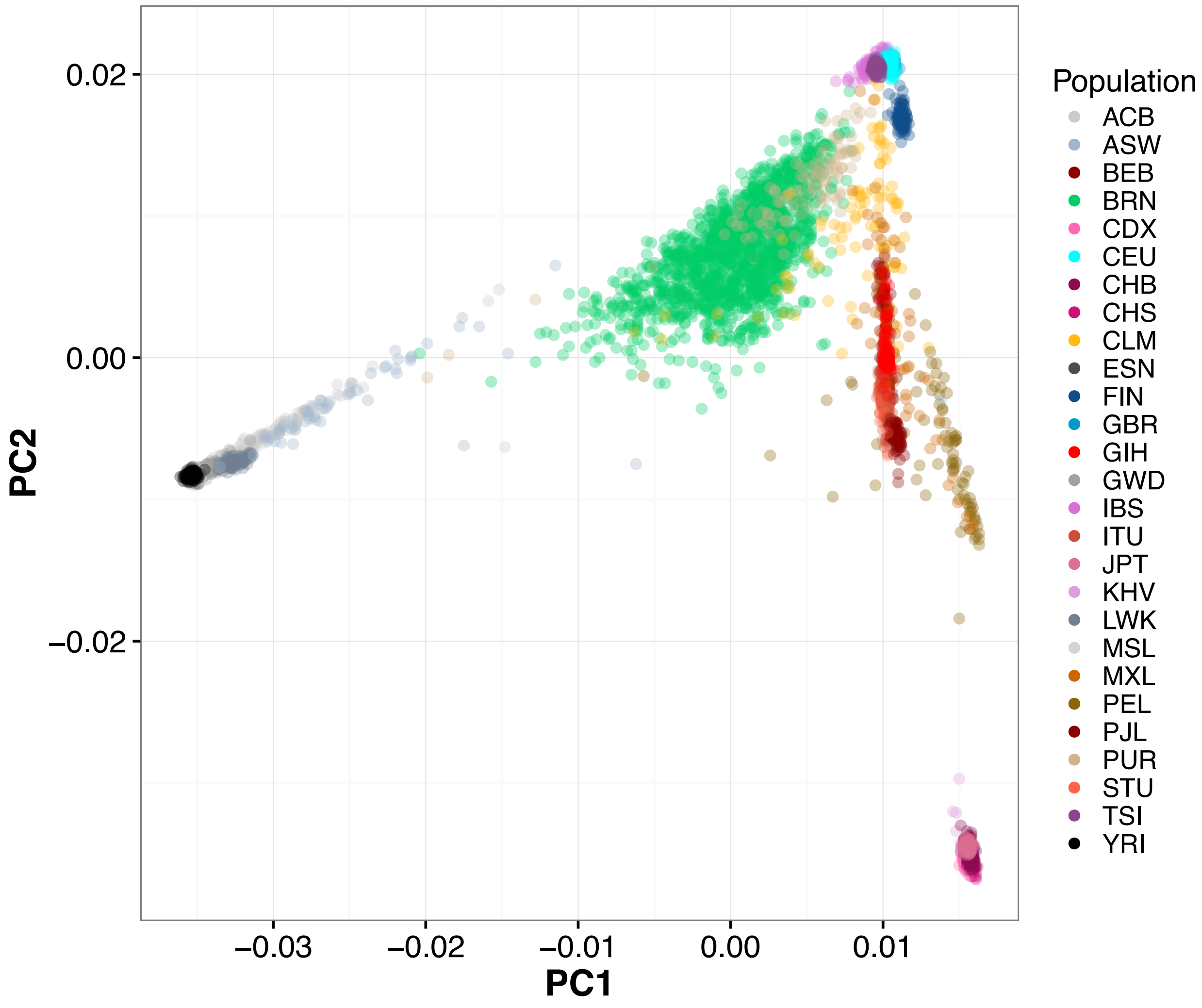

0.02
0.00
−0.02
PC2
−0.03
−0.02
−0.01
0.00
0.01
PC1
Population
ACB
ASW
BEB
BRN
CDX
CEU
CHB
CHS
CLM
ESN
FIN
GBR
GIH
GWD
IBS
ITU
JPT
KHV
LWK
MSL
MXL
PEL
PJL
PUR
STU
TSI
YRI

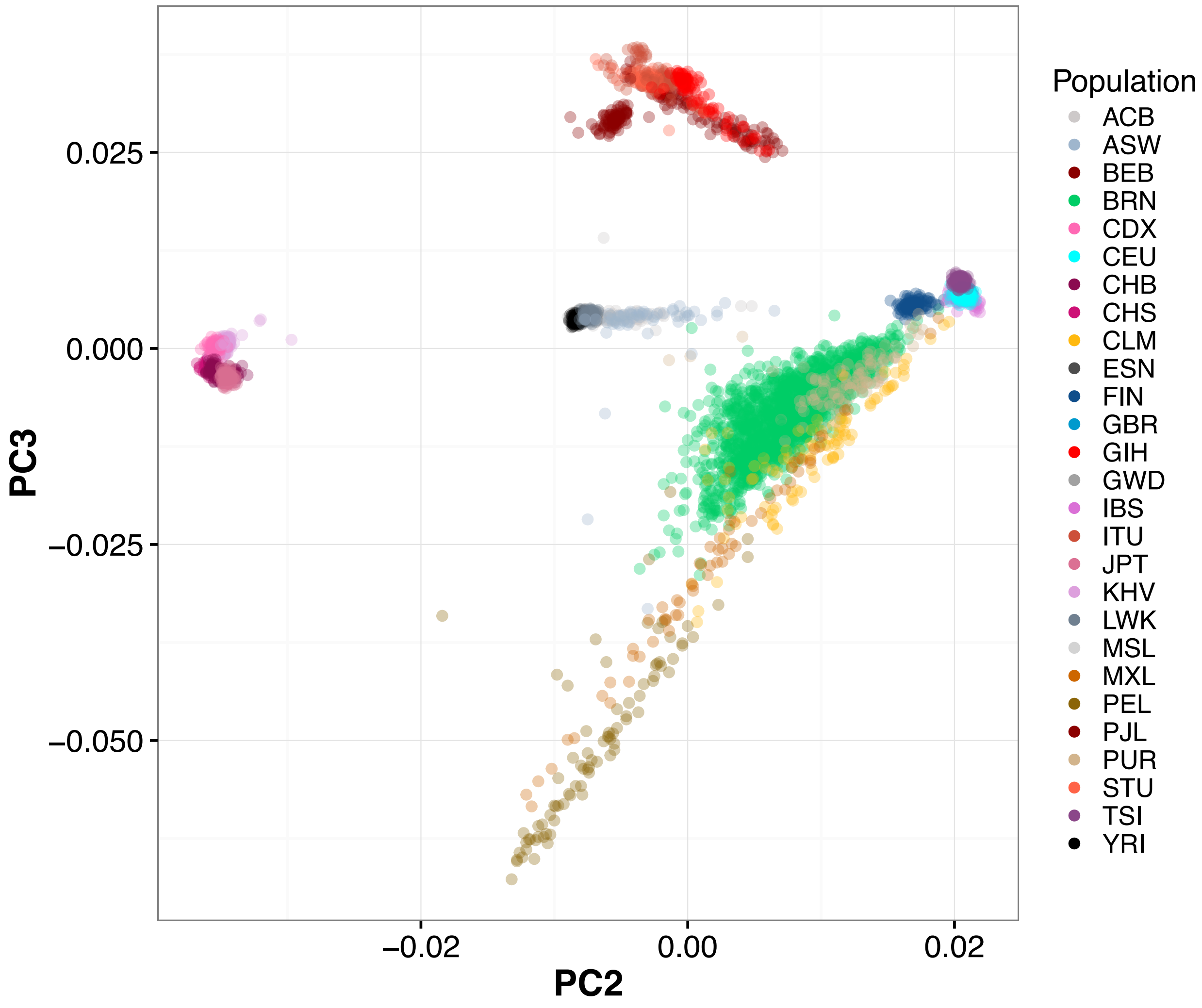

Population
ACB
ASW
BEB
BRN
CDX
CEU
CHB
CHS
CLM
ESN
FIN
GBR
GIH
GWD
IBS
ITU
JPT
KHV
LWK
MSL
MXL
PEL
PJL
PUR
STU
TSI
YRI
PC3
PC2
0.025
0.000
−0.025
−0.050
−0.02
0.00
0.02

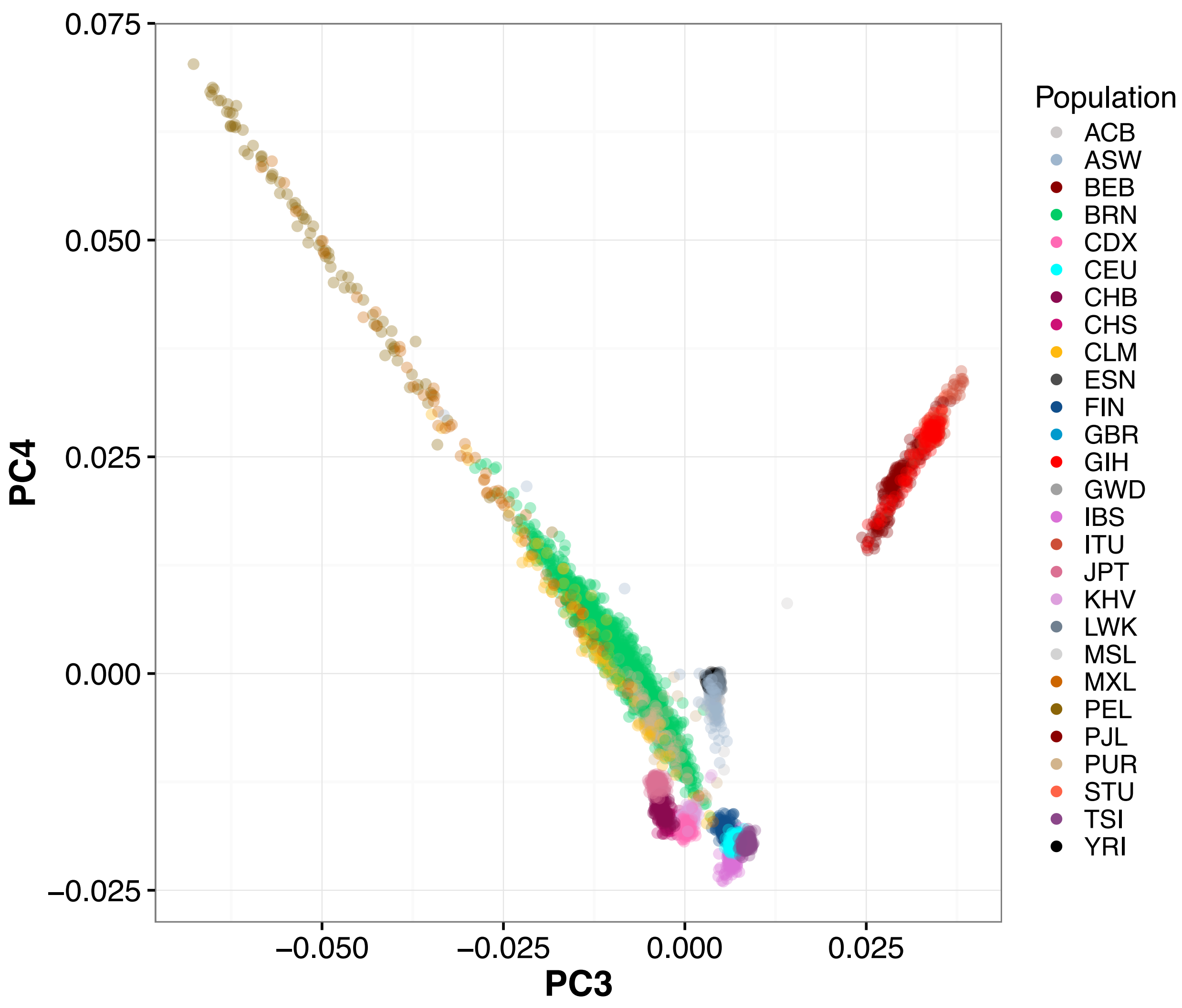
0.075
0.050
0.025
0.000
−0.025
PC4
−0.050
−0.025
0.000
0.025
PC3
Population
ACB
ASW
BEB
BRN
CDX
CEU
CHB
CHS
CLM
ESN
FIN
GBR
GIH
GWD
IBS
ITU
JPT
KHV
LWK
MSL
MXL
PEL
PJL
PUR
STU
TSI
YRI

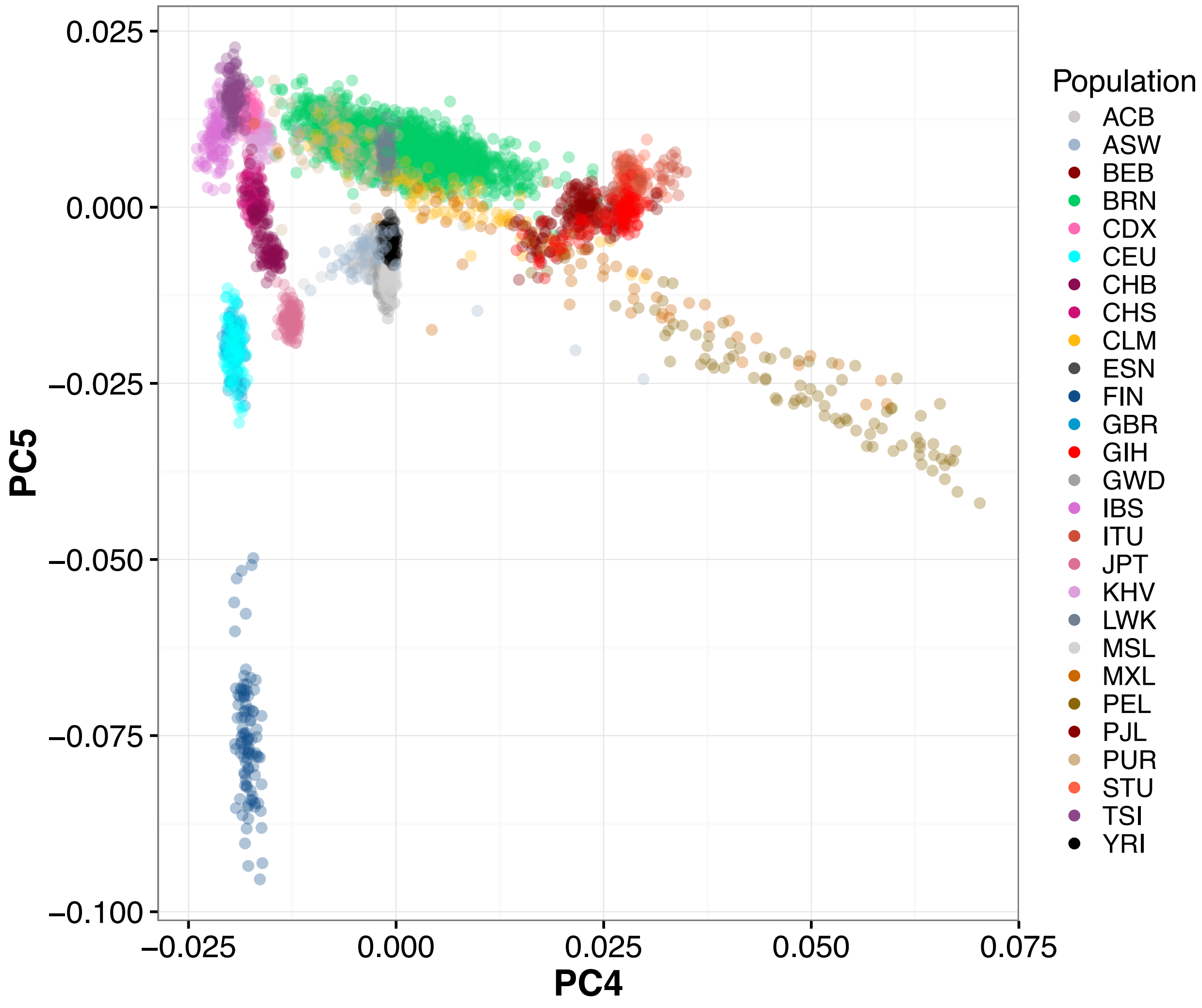
0.025
0.000
−0.025
−0.050
−0.075
−0.100
PC5
−0.025
0.000
0.025
0.050
0.075
PC4
Population
ACB
ASW
BEB
BRN
CDX
CEU
CHB
CHS
CLM
ESN
FIN
GBR
GIH
GWD
IBS
ITU
JPT
KHV
LWK
MSL
MXL
PEL
PJL
PUR
STU
TSI
YRI

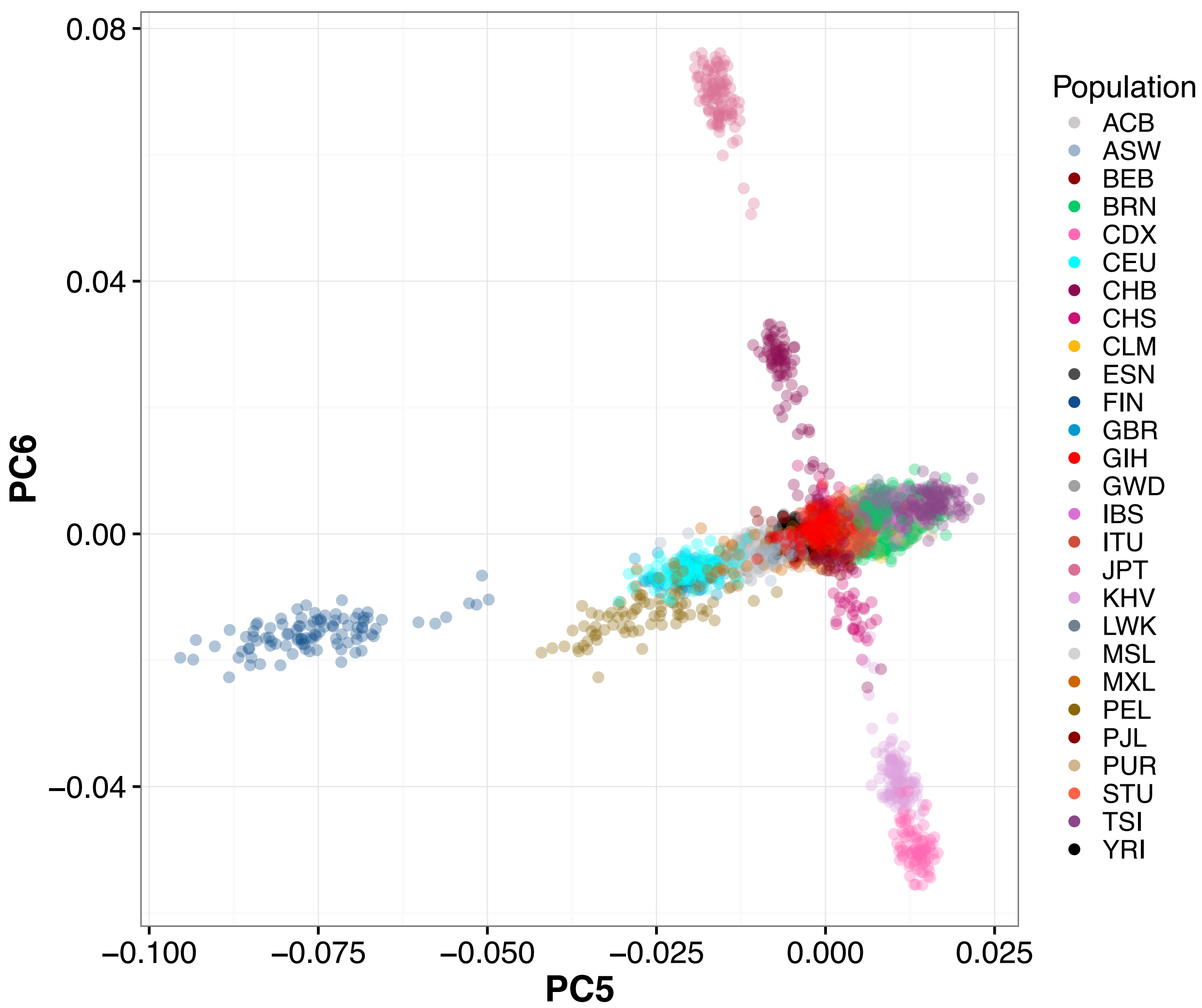
0.08
0.04
0.00
−0.04
PC6
−0.100
−0.075
−0.050
−0.025
0.000
0.025
PC5
Population
ACB
ASW
BEB
BRN
CDX
CEU
CHB
CHS
CLM
ESN
FIN
GBR
GIH
GWD
IBS
ITU
JPT
KHV
LWK
MSL
MXL
PEL
PJL
PUR
STU
TSI
YRI

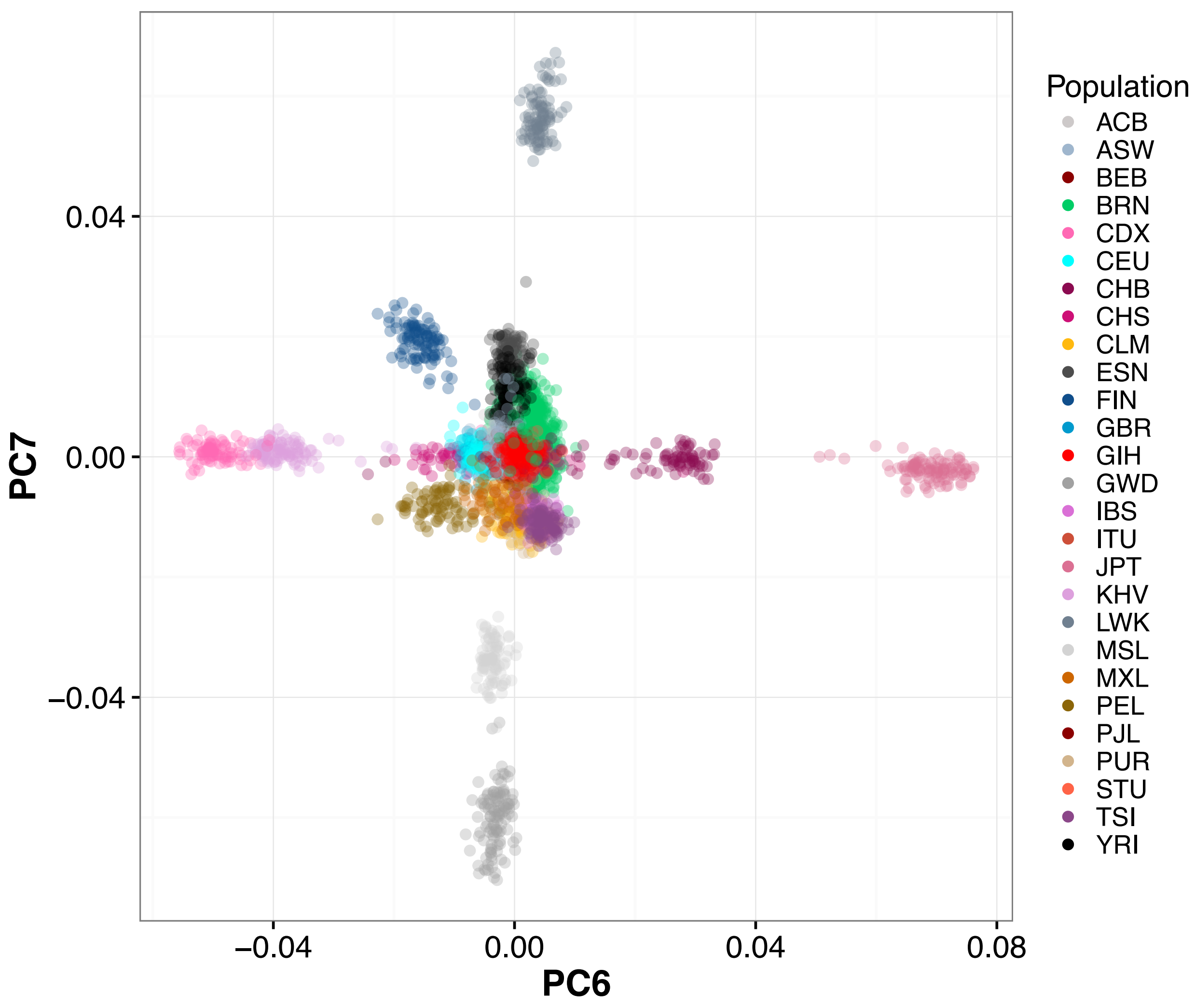

Population
ACB
ASW
BEB
BRN
CDX
CEU
CHB
CHS
CLM
ESN
FIN
GBR
GIH
GWD
IBS
ITU
JPT
KHV
LWK
MSL
MXL
PEL
PJL
PUR
STU
TSI
YRI
PC7
PC6
0.04
0.00
−0.04
−0.04
0.00
0.04
0.08

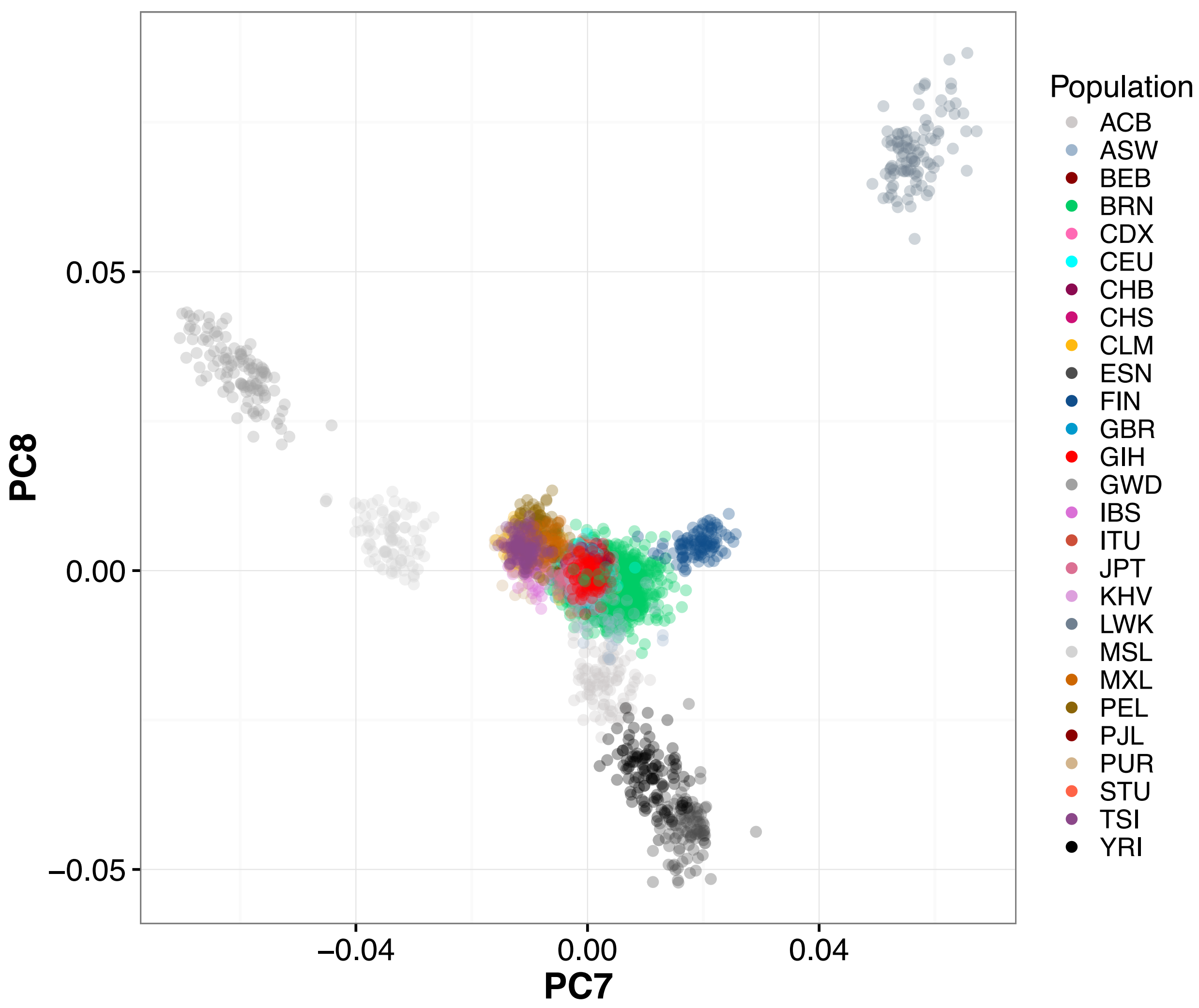

PC8
0.05
0.00
−0.05
−0.04
0.00
0.04
PC7
Population
ACB
ASW
BEB
BRN
CDX
CEU
CHB
CHS
CLM
ESN
FIN
GBR
GIH
GWD
IBS
ITU
JPT
KHV
LWK
MSL
MXL
PEL
PJL
PUR
STU
TSI
YRI

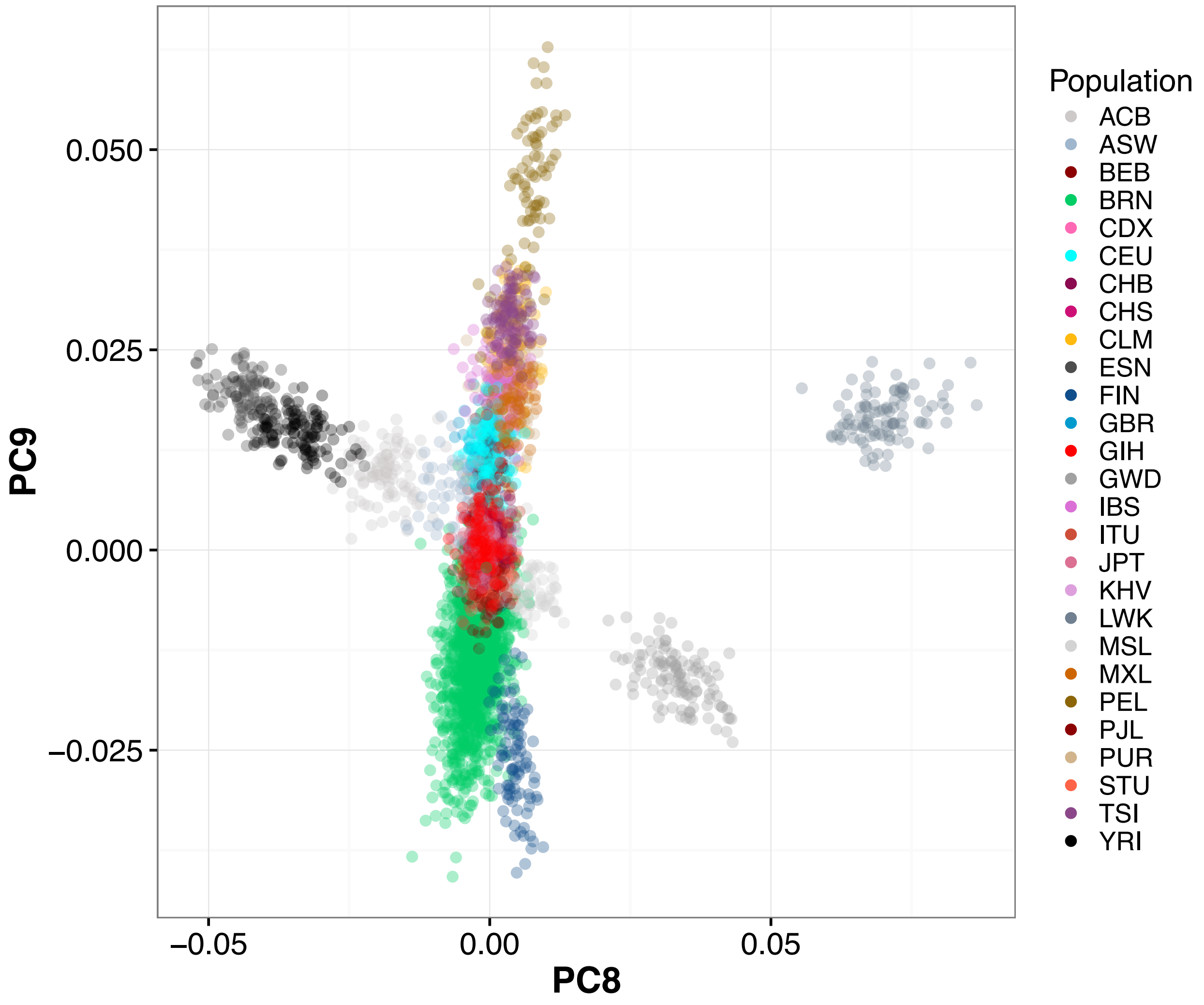

PC9
PC8
0.050
0.025
0.000
−0.025
−0.05
0.00
0.05
Population
ACB
ASW
BEB
BRN
CDX
CEU
CHB
CHS
CLM
ESN
FIN
GBR
GIH
GWD
IBS
ITU
JPT
KHV
LWK
MSL
MXL
PEL
PJL
PUR
STU
TSI
YRI

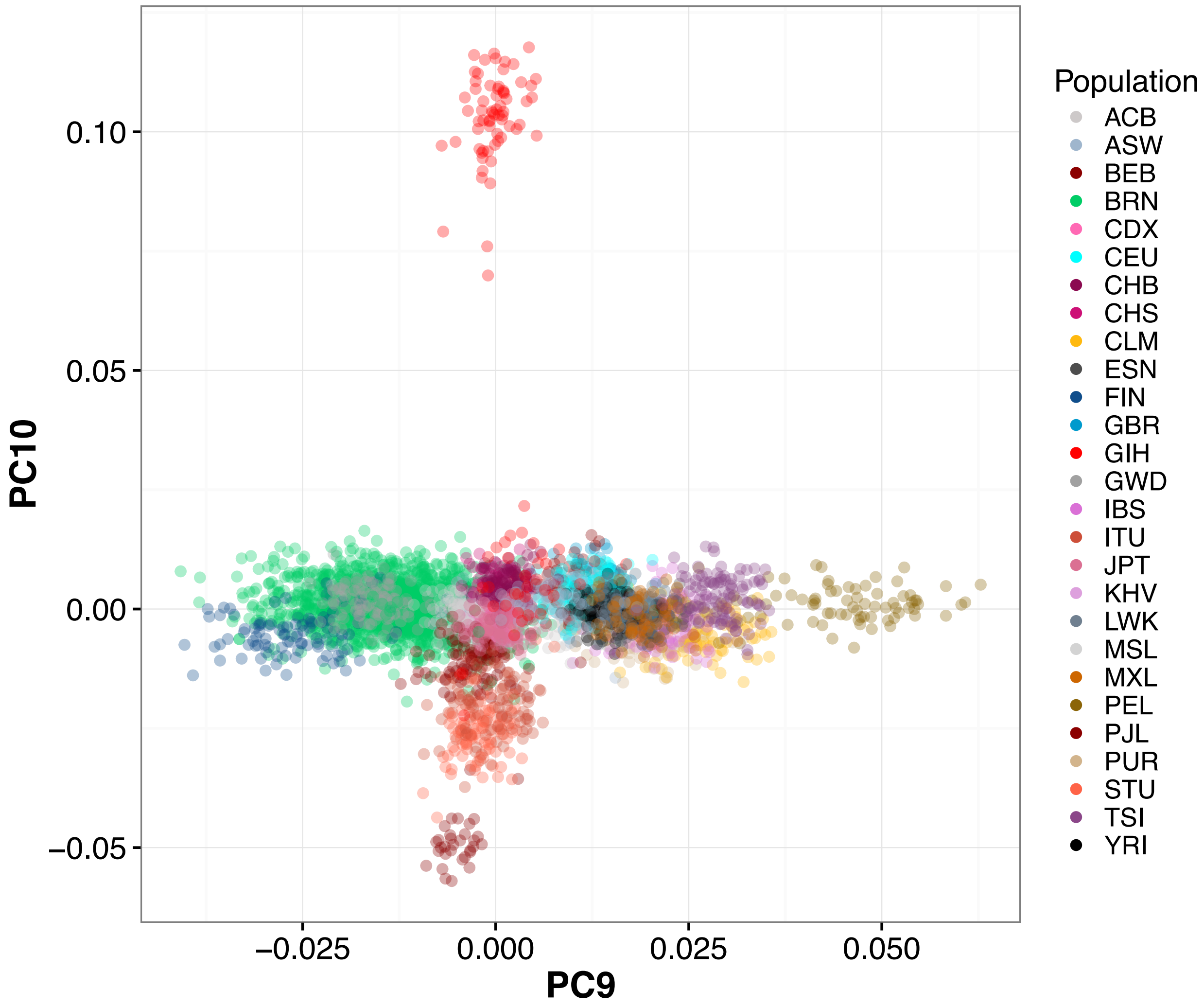
PC10
PC9
0.10
0.05
0.00
−0.05
−0.025
0.000
0.025
0.050
Population
ACB
ASW
BEB
BRN
CDX
CEU
CHB
CHS
CLM
ESN
FIN
GBR
GIH
GWD
IBS
ITU
JPT
KHV
LWK
MSL
MXL
PEL
PJL
PUR
STU
TSI
YRI

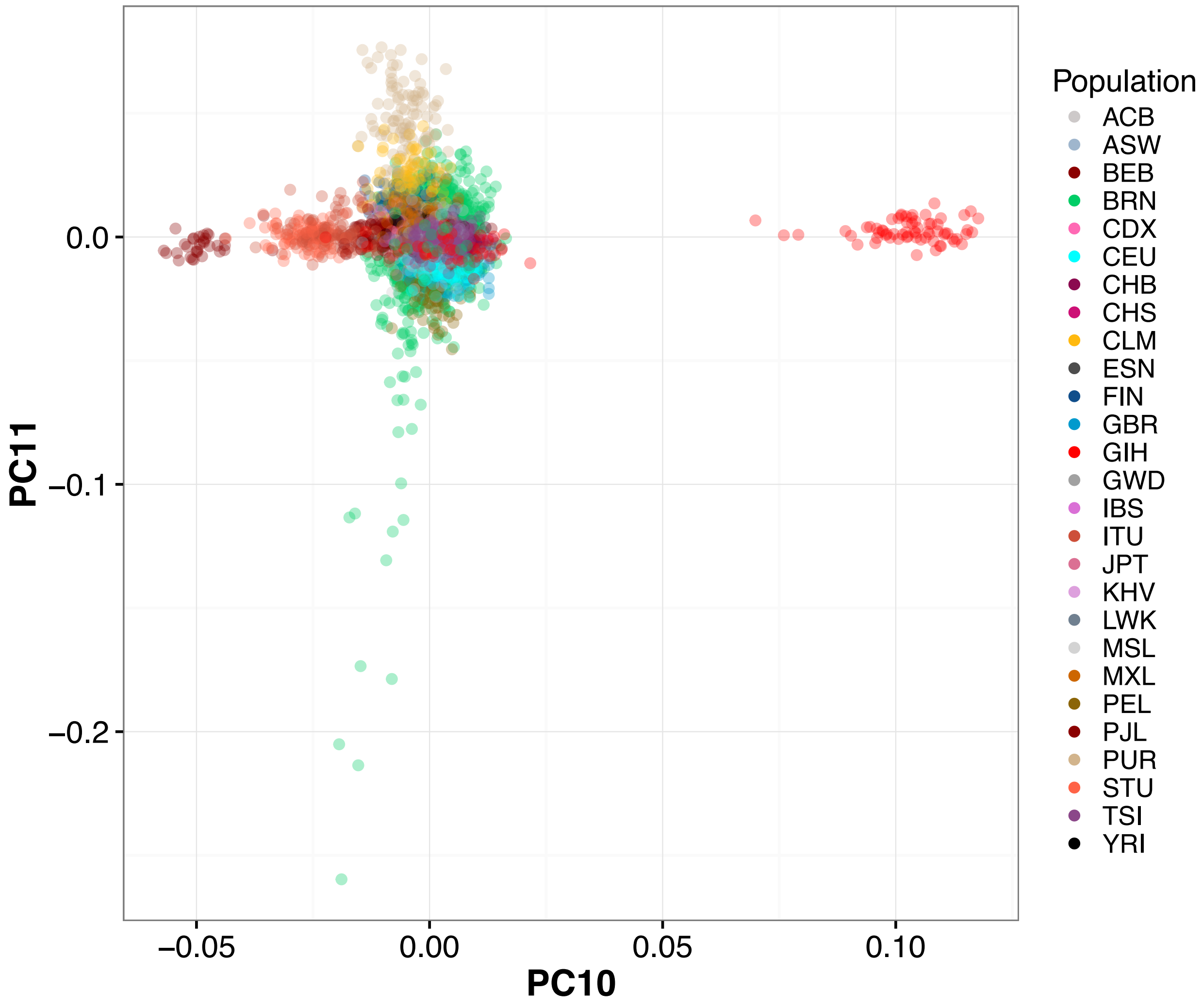
Population
ACB
ASW
BEB
BRN
CDX
CEU
CHB
CHS
CLM
ESN
FIN
GBR
GIH
GWD
IBS
ITU
JPT
KHV
LWK
MSL
MXL
PEL
PJL
PUR
STU
TSI
YRI
PC11
0.0
−0.1
−0.2
PC10
−0.05
0.00
0.05
0.10

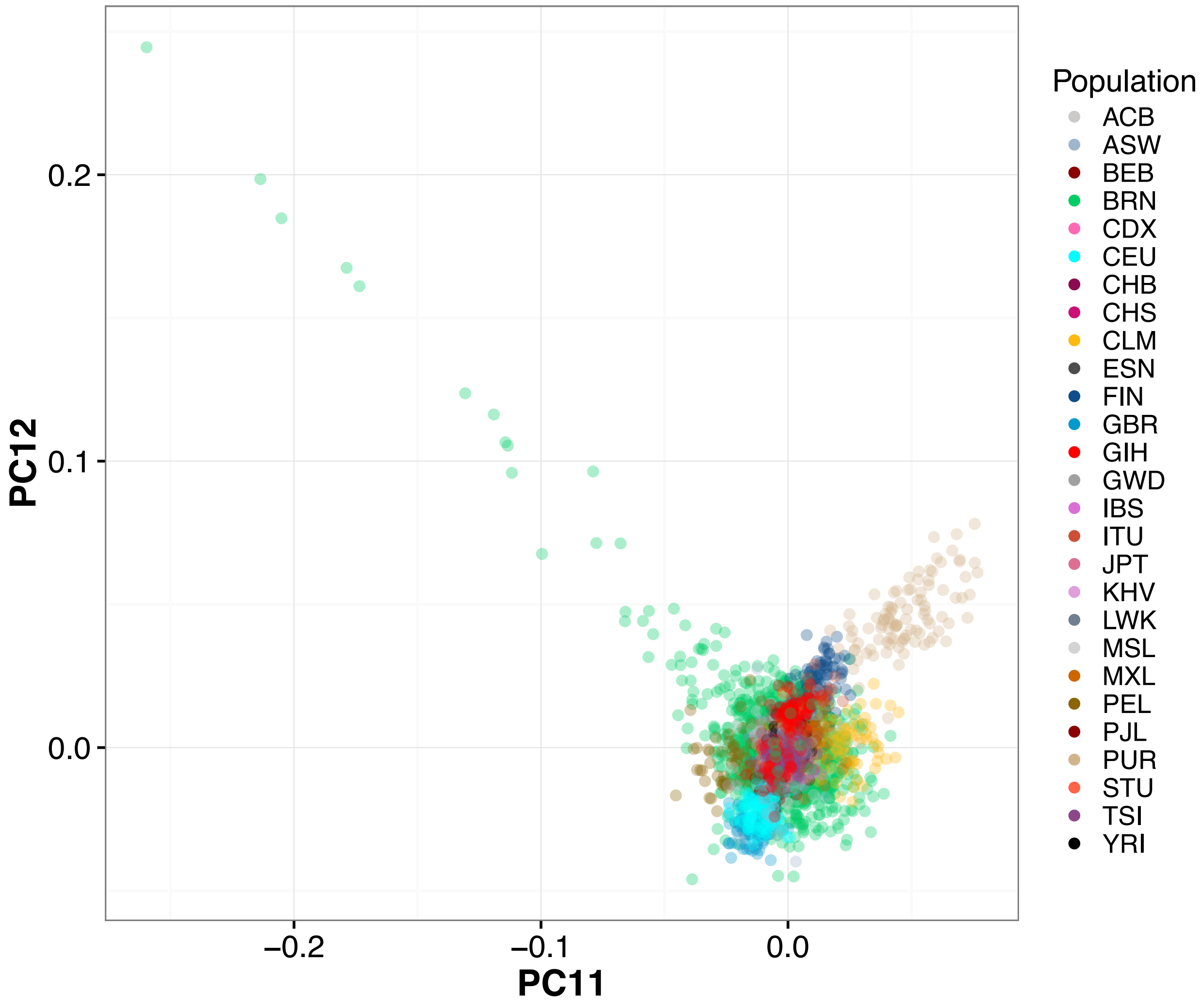

PC12
0.2
0.1
0.0
−0.2
−0.1
0.0
PC11
Population
ACB
ASW
BEB
BRN
CDX
CEU
CHB
CHS
CLM
ESN
FIN
GBR
GIH
GWD
IBS
ITU
JPT
KHV
LWK
MSL
MXL
PEL
PJL
PUR
STU
TSI
YRI

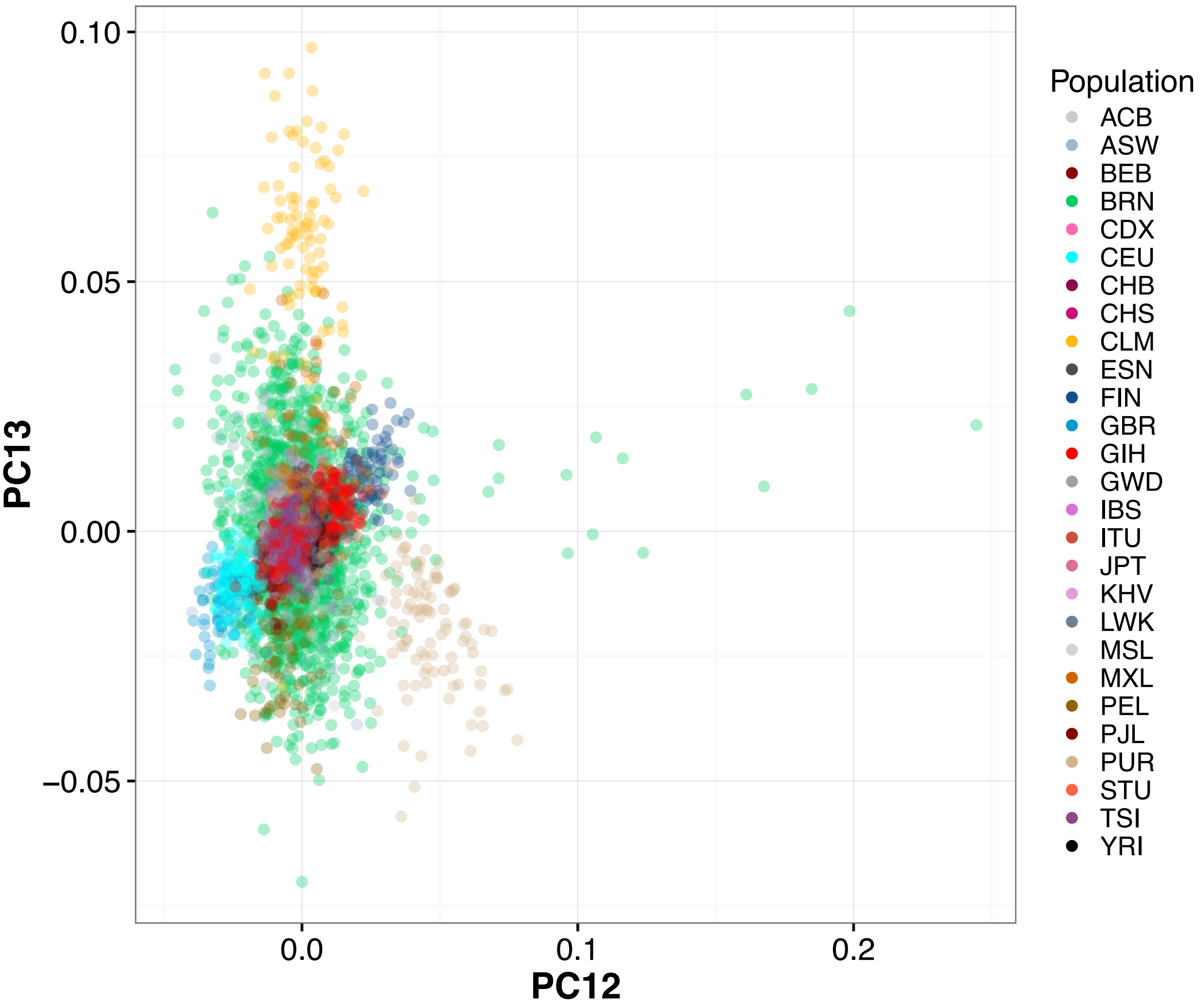
0.10
0.05
0.00
−0.05
PC13
0.0
0.1
0.2
PC12
Population
ACB
ASW
BEB
BRN
CDX
CEU
CHB
CHS
CLM
ESN
FIN
GBR
GIH
GWD
IBS
ITU
JPT
KHV
LWK
MSL
MXL
PEL
PJL
PUR
STU
TSI
YRI

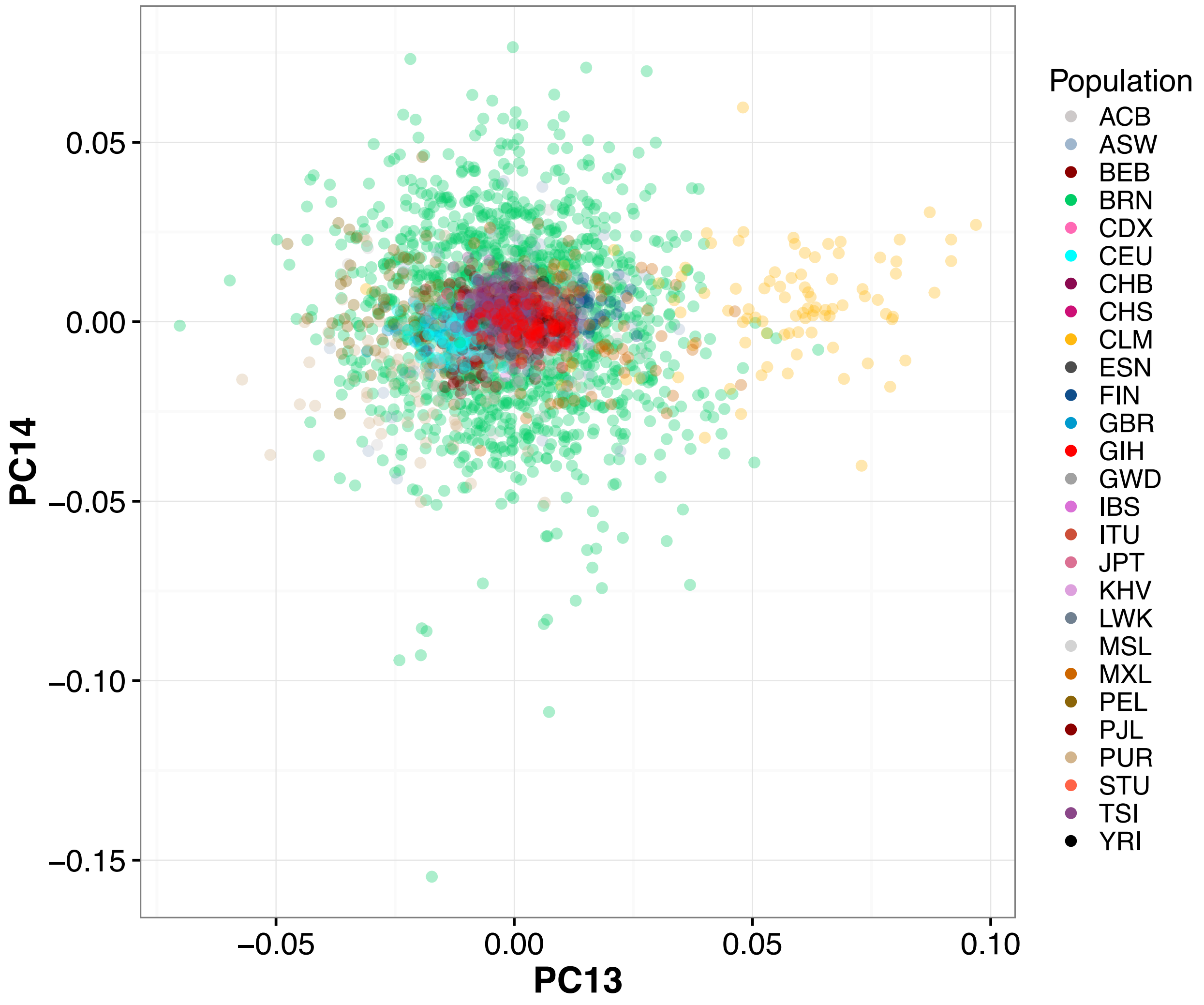

PC14
PC13
0.05
0.00
−0.05
−0.10
−0.15
−0.05
0.00
0.05
0.10
Population
ACB
ASW
BEB
BRN
CDX
CEU
CHB
CHS
CLM
ESN
FIN
GBR
GIH
GWD
IBS
ITU
JPT
KHV
LWK
MSL
MXL
PEL
PJL
PUR
STU
TSI
YRI

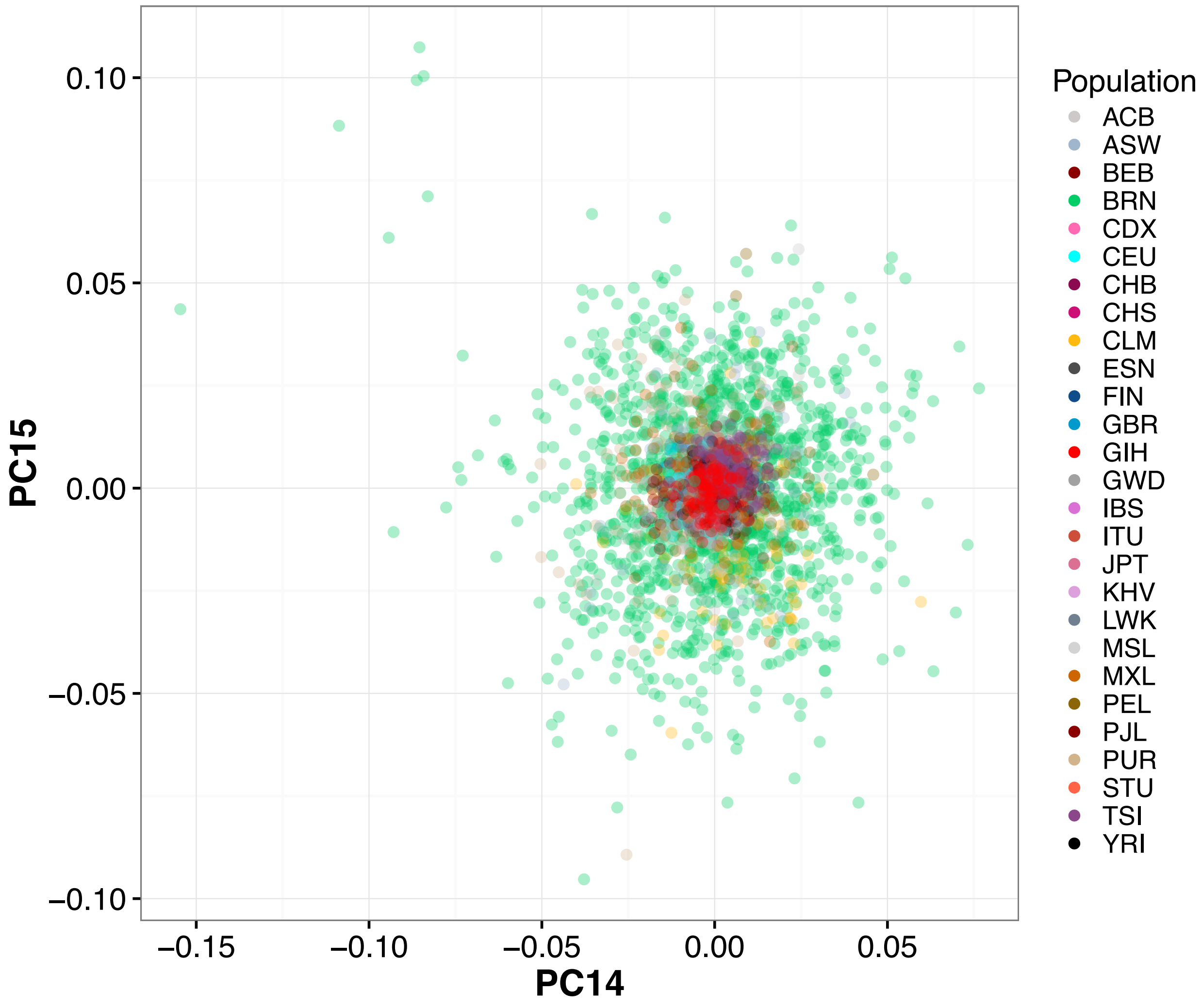

PC15
PC14
0.10
0.05
0.00
−0.05
−0.10
−0.15
−0.10
−0.05
0.00
0.05
Population
ACB
ASW
BEB
BRN
CDX
CEU
CHB
CHS
CLM
ESN
FIN
GBR
GIH
GWD
IBS
ITU
JPT
KHV
LWK
MSL
MXL
PEL
PJL
PUR
STU
TSI
YRI
