# Supplementary material for: Genome-Wide Analysis in Brazilians Reveals Highly Differentiated Native American Genome Regions
Source: Mol Biol Evol. 2017 Jan 18;34(3):559–74. doi: 10.1093/molbev/msw249 (PMC5430616; doi:10.1093/molbev/msw249)
Supplement: Supplementary Data [file msw249_Supp.zip › Supplementary Tables.pdf]

## Supplementary Tables

### Supplementary Table S1. Detailed genome-wide genotyping quality control results for the 6 North-Eastern Brazil study groups

| | Total | Goncalves-Diaz | Mal-ED Birth | Mal-ED Case Control | Recodisa Case Control | PU Zinc-Arginine Trial | PU Zinc-Vitamin A Trial |
|---|---|---|---|---|---|---|---|
| **Samples Genotyped** | 2,119 | 172 | 300 | 368 | 1044 | 126 | 109 |
| | | | | | | | |
| **Affymetrix QC Criterion**[a] | | | | | | | |
| Dropped DQC < 0.82 | -238 | -2 | -5 | -12 | -212 | -1 | -6 |
| Dropped Call Rate < 97% | -192 | 0 | -8 | -18 | -157 | -3 | -6 |
| **Post-Affymetrix Axiom QC**[b] | **1,689** | **170** | **287** | **338** | **675** | **122** | **97** |
| Dropped Mismatch sex; duplicates | -30 | -1 | -6 | -2 | -12 | -6 | -3 |
| Dropped Related up to degree 2 | -121 | -59 | -5 | 0 | -5 | -21 | -31 |
| **Post-Genotype QC** | **1,538** | **110** | **276** | **336** | **658** | **95** | **63** |
| | | | | | | | |
| **SNP QC** | | | | | | | |
| Initial SNPs on Affymetrix Axiom LAT-1 Array 4 | 818,154 | | | | | | |
| Affymetrix SNP QC: SNPs dropped | -62,353 | | | | | | |
| **SNPs Remaining** | **755,801** | | | | | | |

[a] Standard Affymetrix Axiom genome-wide sample QC metrics: Dish QC (DQC) < 0.82; Sample Genotype Call Rate < 97%.
[b] Combined Post-Affymetrix Axiom QC identified 30 total samples to drop because either:
1) Mismatch between inferred sex and study database sex; 2) Mislabeled duplicate samples;
and a further 121 to drop because of intra-and inter-study relatedness up to second degree.

Standard Affymetrix Axiom genome-wide SNP QC was applied using SNPolish R script (Affymetrix).

**Supplementary Table S2. 1000 Genomes Populations in the 2013/05/02 Release**

| Population | Country of Origin | Code |
|---|---|---|
| *East Asian* | | |
| Chinese Dai in Xishuangbanna | China | CDX |
| Han Chinese in Bejing | China | CHB |
| Japanese in Tokyo | Japan | JPT |
| Kinh in Ho Chi Minh City | Vietnam | KHV |
| Southern Han Chinese | China | CHS |
| | | |
| *South Asian* | | |
| Bengali in Bangladesh | Bangladesh | BEB |
| Gujarati Indian in Houston, TX | USA | GIH |
| Indian Telugu in the UK | UK | ITU |
| Punjabi in Lahore, Pakistan | Pakistan | PJL |
| Sri Lankan Tamil in the UK | UK | STU |
| | | |
| *African* | | |
| African Ancestry in Southwest US | USA | ASW |
| African Caribbean in Barbados | Barbados | ACB |
| Esan in Nigeria | Nigeria | ESN |
| Gambian in Western Division | The Gambia | GWD |
| Luhya in Webuye | Kenya | LWK |
| Mende in Sierra Leone | Sierra Leone | MSL |
| Yoruba in Ibadan | Nigeria | YRI |
| | | |
| *European* | | |
| British in England and Scotland | UK | GBR |
| Finnish in Finland | Finland | FIN |
| Iberian populations in Spain | Spain | IBS |
| Toscani in Italy | Italy | TSI |
| Utah residents with Northern and Western European ancestry | USA | CEU |
| | | |
| *Americas* | | |
| Colombian in Medellin | Colombia | CLM |
| Mexican Ancestry in Los Angeles | USA | MXL |
| Peruvian in Lima | Peru | PEL |
| Puerto Rican in Puerto Rico | Puerto Rico | PUR |

**Supplementary Table S3. Selected proxy samples required for supervised ADMIXTURE analysis**

| Total Number of Amerindian Ancestry Proxy Samples | PEL | MXL | CLM | ASW |
|---|---|---|---|---|
| N=0 | - | - | - | - |
| N=10 | 10 | - | - | - |
| N=20 | 19 | 1 | - | - |
| N=30 | 27 | 3 | - | - |
| N=40 | 36 | 4 | - | - |
| N=50 | 44 | 6 | - | - |
| N=100 | 76 | 21 | 2 | 1 |
| | | | | |
| **Total Number of African Ancestry Proxy Samples** | **ESN** | **YRI** | **MSL** | |
| N=0 | - | - | - | |
| N=10 | 5 | 5 | - | |
| N=20 | 12 | 8 | - | |
| N=30 | 18 | 12 | - | |
| N=40 | 22 | 18 | - | |
| N=50 | 30 | 20 | - | |
| N=100 | 55 | 43 | 2 | |

The table shows the total number of samples selected from each of the 1KG Latin America populations based on principal component analysis (Figure 2 and main text) for each total number of Amerindian proxy samples required for supervised ADMIXTURE analysis. Similarly for the African ancestry proxies.

**Supplementary Table S4. Supervised ancestry estimates by study group.**

| **Study Group** | **Enrolled from** | **N (post-QC)** | **% EUR Ancestry mean ± stddev** | **% AMR Ancestry mean ± stddev** | **% AFR Ancestry mean ± stddev** |
|---|---|---|---|---|---|
| Goncalves-Diaz | Fortaleza | 110 | 53.2 ± 11.0 | 24.6 ± 7.2 | 22.2 ± 7.8 |
| Mal-ED Birth | Fortaleza | 276 | 54.5 ± 9.9 | 23.8 ± 6.2 | 21.8 ± 6.6 |
| Mal-ED Case Control | Fortaleza | 336 | 52.3 ± 10.0 | 25.5 ± 6.6 | 22.5 ± 6.2 |
| Recodisa Case Control | 6 Cities, 4 North-Eastern States | 658 | 61.2 ± 11.8 | 14.6 ± 4.3 | 24.2 ± 9.2 |
| PU Zinc-Arginine Trial | Fortaleza | 95 | 54.9 ± 8.9 | 23.5 ± 5.3 | 21.6 ± 5.7 |
| PU Zinc Vitamin A Trial | Fortaleza | 63 | 54.3 ± 11.1 | 25.2 ± 7.0 | 20.6 ± 6.3 |
| **Total** | | **1,538** | **56.8 ± 11.5** | **20.3 ± 7.5** | **22.9 ± 7.8** |

The study groups are as described in Table 1 of the main text and the genotyping summaries in Supplementary Table S1.

Hotelling's 2 df test was applied to each pair of study groups testing equality of means of (%AMR, %AFR)

The means of Recodisa Case Control samples were significantly different from all 5 other study groups ($p<1x10^{-10}$) but no other pair of groups was different after correction for multiple testing (15 tests).

**Supplementary Table S5. Fst values for the pairwise comparison of the Amerindian (second) admixture component in Brazil samples (BRN2 (Amr)) versus 1KG Asian populations, clustered by degree of differentiation**

| **1KG Asian Population** | **Asian Region** | **Fst BRN2 (Amr) vs 1KG** | **Bootstrap Percentile 95% CI** |
|---|---|---|---|
| | | | |
| *Cluster 1* | | | |
| BEB, Bengali | South | 0.0986 | ( 0.0979, 0.0993 ) |
| | | | |
| *Cluster 2* | | | |
| PJL, Punjabi | South | 0.1030 | ( 0.1024, 0.1037 ) |
| CHB, Han Chinese | East | 0.1033 | ( 0.1025, 0.1040 ) |
| JPT, Japanese | East | 0.1035 | ( 0.1028, 0.1043 ) |
| | | | |
| *Cluster 3* | | | |
| STU, Sri Lanka Tamil | South | 0.1061 | ( 0.1054, 0.1067 ) |
| CHS, Southern Han Chinese | East | 0.1062 | ( 0.1054, 0.1069 ) |
| GIH, Gujarati | South | 0.1067 | ( 0.1060, 0.1074 ) |
| ITU, Indian Telugu | South | 0.1067 | ( 0.1060, 0.1074 ) |
| KHV, Kinh | East | 0.1074 | ( 0.1066, 0.1081 ) |
| | | | |
| *Cluster 4* | | | |
| CDX, Chinese Dai | East | 0.1110 | ( 0.1102, 0.1118 ) |

Clusters are ranked from lowest to highest genetic differentiation compared to the Amerindian admixture component in Brazil and defined by the maximal 95% bootstrap percentile of all members. Bootstrap percentiles are from 10,000 resamples of 195,090 loci.
